# Maternal Bisphenol A Exposure Impacts the Fetal Heart Transcriptome

**DOI:** 10.1371/journal.pone.0089096

**Published:** 2014-02-25

**Authors:** Kalyan C. Chapalamadugu, Catherine A. VandeVoort, Matthew L. Settles, Barrie D. Robison, Gordon K. Murdoch

**Affiliations:** 1 Department of Animal and Veterinary Science, University of Idaho, Moscow, Idaho, United States of America; 2 Department of Obstetrics and Gynecology, University of California Davis, Davis, California, United States of America; 3 Department of Computer Science, University of Idaho, Moscow, Idaho, United States of America; 4 Department of Biological Sciences, University of Idaho, Moscow, Idaho, United States of America; 5 Program in Bioinformatics and Computational Biology, University of Idaho, Moscow, Idaho, United States of America; 6 California National Primate Research Center, University of California Davis, Davis, California, United States of America; University of Hyderabad, India

## Abstract

Conditions during fetal development influence health and disease in adulthood, especially during critical windows of organogenesis. Fetal exposure to the endocrine disrupting chemical, bisphenol A (BPA) affects the development of multiple organ systems in rodents and monkeys. However, effects of BPA exposure on cardiac development have not been assessed. With evidence that maternal BPA is transplacentally delivered to the developing fetus, it becomes imperative to examine the physiological consequences of gestational exposure during primate development. Herein, we evaluate the effects of daily, oral BPA exposure of pregnant rhesus monkeys (*Macaca mulatta*) on the fetal heart transcriptome. Pregnant monkeys were given daily oral doses (400 µg/kg body weight) of BPA during early (50–100±2 days post conception, dpc) or late (100±2 dpc – term), gestation. At the end of treatment, fetal heart tissues were collected and chamber specific transcriptome expression was assessed using genome-wide microarray. Quantitative real-time PCR was conducted on select genes and ventricular tissue glycogen content was quantified. Our results show that BPA exposure alters transcription of genes that are recognized for their role in cardiac pathophysiologies. Importantly, myosin heavy chain, cardiac isoform alpha (*Myh6*) was down-regulated in the left ventricle, and ‘A Disintegrin and Metalloprotease 12’, long isoform (*Adam12-l*) was up-regulated in both ventricles, and the right atrium of the heart in BPA exposed fetuses. BPA induced alteration of these genes supports the hypothesis that exposure to BPA during fetal development may impact cardiovascular fitness. Our results intensify concerns about the role of BPA in the genesis of human metabolic and cardiovascular diseases.

## Introduction

Bisphenol A (BPA) is used in the manufacture of polycarbonate plastics and epoxy resins that line food and beverage containers and a wide variety of consumer products such as water bottles and sales receipts; this results in repetitive, long-term exposure in most humans. BPA exposure in pregnant women can cross transplacentally, potentially impacting the developing fetus [1], [2]. While the adult conjugates BPA into BPA-glucuronide (BPA-GA) in the liver for excretion in the bile, this metabolic pathway is quite low in the fetus; of greater concern, BPA-GA is deconjugated in the fetus increasing exposure to the more biologically potent BPA [2]. Furthermore, BPA can interact with several biologically important hormone receptors; estrogen [3], [4], androgen [5], thyroid [6], [7] and intercellular communication channels such as CX-43 based gap junctions [8], though it remains unclear whether the associated signaling cascades are activated or inhibited. There is growing evidence that the receptor-mediated responses to BPA are dependent upon dose, duration and timing of BPA exposure as well as the cell/tissue studied [9]–[12].

The Barker hypothesis, first presented to explain the increased risk of cardiovascular disease in children of undernourished mothers of the Dutch famine, suggests that fetal developmental “programming” predisposes some individuals to the development of chronic disease later in life. The crux of this theory is that the phenotype of an individual is dependent upon both genotype and environmental influences that alter prenatal growth rate, temporal growth patterns and birth weight [13]. Most relevant to this work is that Barker extended this theory by directly linking the incidence of cardiovascular disease and insulin resistance to the placental environment [14]. While the BPA specific effects on adults is widely acknowledged in animal studies [15]–[17], fetal BPA exposure also affects multiple organ systems, with behavioral, reproductive and metabolic consequences in the adult [18]. Recent evidence also show that exposure to BPA during pregnancy in mice not only alters glucose homeostasis but also serves as a risk factor for subsequent development of diabetes and heart disease in the offspring [19]. Intriguingly, epidemiological studies also suggest a correlation between higher urinary BPA concentrations in adult men and increased incidence of cardiovascular disease and diabetes [20]–[22]

Cardiovascular disease and diabetes are chronic illnesses that are rising in incidence and are among the top 5 diseases globally in terms of burden on the health care system. Cardiac tissue is known to express estrogen [23], androgen [24] and thyroid receptors [25], [26] and these hormones play critical roles in the normal developmental process of cardiac tissue; as such we investigate the potential for disruption of these developmental mediators by exposure to BPA. The objective of this study was to evaluate the effects of daily, oral maternal BPA exposure in primates on the gene expression profiles in each of the left ventricle (LV), right ventricle (RV), left atrium (LA) and right atrium (RA) of developing fetal heart tissue. This study is the first report of the impact of maternal BPA exposure on fetal/neonatal cardiovascular tissue in a primate.

## Materials and Methods

### Ethics statement

All animal procedures were approved in advance by the Institutional Animal Use and Care Advisory Committee at the University of California, Davis, and all procedures complied with the requirements of the Animal Welfare Act.

### Animal experimentation and tissue collection

The tissues evaluated in this study were part of a study to determine the effects of BPA on ovarian development and detailed information about animal care and BPA exposure have been reported [27]. Therefore, all analysis reported in this study were from female fetal heart tissue. All animal procedures were approved in advance by the Institutional Animal Use and Care Advisory Committee at the University of California, Davis and all procedures complied with the requirements of the Animal Welfare Act. Adult female rhesus monkeys (*Macaca mulatta*) were housed at California National Primate Research Center and maintained according to the institutional guidelines (http://www.cnprc.ucdavis.edu) as previously described [27]–[29]. Animals were housed individually in stainless steel cages and maintained under a 0600–1800 hours light cycle at 25–27°C. All animals were allowed ad libitum access to Purina Monkey Chow and water supplied through PVC pipes and a “lixit” device. Further, environmental enrichment was evoked by offering supplemental seasonal produce, seeds and cereals. Animals with a history of prior pregnancy were bred using established time-mated procedures and pregnancy was detected by ultrasound [30], [31]. Pregnant monkeys were sonographically screened early in gestation to confirm normal development and to identify female fetuses (by 40 days gestation) using established protocols with proven reliability [30], [32]. Fetuses were delivered vaginally or through a caesarian section. Fetal tissues were collected at necropsy and euthanasia conformed to the recommendations of the American Veterinary Medical Association (AVMA) Guidelines on Euthanasia and Primate Center SOPs (overdose of pentobarbital).

The BPA dose selected for this study was determined to lead to peak blood levels in non-pregnant monkeys [28] that approximate those levels that are commonly measured in humans [33]. Pregnant females were administered a small piece of fruit once daily that contained either deuterated BPA (400 µg/kg body weight), dissolved in 150 µl ethanol (vehicle) or 150 µl ethanol only (control), during either early gestation, EG (days; 50–100±2; n = 5) or late gestation, LG (days; 100±2 – term; control, *n = *5 and BPA, *n = 4*) [28]. Quantification of maternal serum BPA indicate peak levels of conjugated BPA of 141.08±26.64 ng dBPA/ml and unconjugated BPA of 1.99±0.12 ng dBPA/ml in the EG timeline. In the LG time line, the maternal serum conjugated and unconjugated BPA levels were 39.09±15.71 ng/ml and 0.68±0.312 ng/ml, respectively [29]. As explained earlier, fetal heart tissues of left ventricle (LV), right ventricle (RV), left atrium (LA) and right atrium (RA) were obtained from fetuses removed by caesarian section (EG; control, *n = 5* & BPA, *n = 4*) or at necropsy following vaginal delivery (LG; control, *n = 5* & BPA, *n = 4* ). Other fetal tissues were harvested from these fetuses and results of their analyses have been reported previously [27], [29].

### RNA isolation

Total RNA was extracted from rhesus monkey fetal LV, RV, LA and RA tissue using TRIzol reagent (Invitrogen, Carslbad, CA, USA) according to the manufacturer's protocol. RNA was quantified using Nanodrop® ND-1000 UV–Vis Spectrophotometer (Nanodrop Technologies, Wilmington, DE, USA) and integrity of RNA was assessed using 1.5% denaturing formaldehyde agarose gel electrophoresis. Subsequently, 15 µg of DNase treated (Ambion, Foster City, CA, USA) total RNA was utilized for cDNA synthesis.

### Custom NimbleGen Rhesus Microarray design

A custom unbiased NimbleGen rhesus macaque 12-plex whole transcript gene expression microarray (Roche NimbleGen, Madison, WI, USA) was designed from the European Bioinformatics Institute (EBI) Ensembl database (*Macaca mulatta*, Mmul_1, Jan 2006). We identified gene transcripts (21,905) and small RNAs (5,253) from the entire genome (total 28,920 potential transcripts). Of the 21,905 transcripts targeted, we were able to build probes for 21,840 of which 21,808 contained 6 probes each and the rest (32) had less than 6 probes. Of 5,253 sRNA targeted, we were able to build probes for 4,309 of which 1,974 had a single probe and 2,335 contained 2 probes for a total of 137,577 unique probes (60 ‘mer in length) on the array.

### Microarray sample preparation, hybridization and scanning

Briefly, 10 µg of DNase treated total RNA from LV or RV was primed with 100 picomoles of oligo (dT_18_) and 200 ng of random primers and reverse transcribed using 1.5 µL of superscript III (Invitrogen). Second strand cDNA was synthesized and RNase treated to remove residual RNA. Due to the limitations in the quantity of tissue available from the LA and RA, cDNA preparations from both these tissue were prepared by employing a PCR based amplification approach available through TransPlex® Complete Whole Transcriptome Amplification Kit (WTA2) (Sigma, St. Louis, MO, USA). In this case, double stranded cDNA (dscDNA) was synthesized from a 40 ng of DNase treated total RNA from each sample. In both cases, resultant dscDNA was purified by employing phenol:chloroform:isoamyl alcohol, ethanol precipitation and quantified. Finally, cDNA was labeled using Cy5 nanomers (NimbleGen). One 12-plex array that contained 12 sub-arrays was used per tissue (LV, RV, LA & RA) to hybridize three samples (control, *n = 3* & BPA, *n = 3*) from each of the BPA or control treatment groups, in both the EG and LG time lines (2 (LG, EG) x 2 (control, BPA) x 3 (replicates)  = 12 samples). Labeled cDNA (2 µg) from each sample was hybridized overnight at 42°C using the NimbleGen Maui Hybridization system 4 (NimbleGen). Arrays were washed using a Maui Wash system (BioMicro Systems, Salt Lake city, UT, USA). Genepix pro 6.1 software (Molecular devices, Downingtown, PA, USA) operating a Genepix4000B scanner (Molecular devices) was used to scan all hybridized arrays. Scanning was performed at a resolution of 5 µm, and photomultiplier tube (PMT) gains were set to obtain maximum signal intensities such that approximately 5% of the probes reached signal saturation (2^16^ raw signal intensity).

### Quantitative real-time Polymerase chain reaction (qRT-PCR)

DNase treated total RNA (2 µg) from each tissue sample of both EG (control, *n = 5* & BPA, *n = 5*) and LG (control, *n = 5* and BPA, *n = 4*) timeline was reverse transcribed using high-capacity cDNA reverse transcription kit (Applied Biosystems, Foster City, CA, USA). Gene expression was measured by employing custom designed gene specific Taqman MGB® primer/probes on an ABI 7500 fast real-time PCR system (Applied Biosystems). Primers (Invitrogen) and probes (Applied Biosystems) (Table 1) were designed to span more than one exon of a gene, using Primer Express 3.0 (Applied Biosystems). Real-time PCR assays were conducted as reported previously [34]. All genes were assayed in duplicate. Endogenous reference, 18 S *rRNA* was measured using 0.5 ng cDNA, and all other genes were assayed using 50 ng cDNA in each reaction.

**Table 1:** Taqman MGB primer & probe system.

Gene	Gene accession number	Primer & Taqman Probe sequences
18 s rRNA	AF243428	FP: CCACGCGAGATTGAGCAAT
		RP: GCAGCCCCGGACATCTAA
		TP: ACAGGTCTGTGATGCC
*Myh6*	ENSMMUT00000020702	FP: AGCGGCGCATCAAGGA
		RP: CGCAGCAGGTTCTTTTTGTCT
		TP: CTCACCTACCAGACAGAG
*Adam12-l*	ENSMMUT00000013487	FP: CGGCCACCTCGGAAAAG
		RP: CCTTCGGTGGGTAGGAATCTG
		TP: CCTGATGAGGAAGCC
(*Thrb*)	ENSMMUT00000000096	FP: TGAATCAATACAGCCAACCTGAA
		RP: GGCGACTGCACTTGAGAAAAA
		TP: ATTTCACTGAAGAAAAGC
*Gyg1*	ENSMMUT00000007776	FP: CTACTCCTCAGGTCTCAGACTCCAT
	ENSMMUT00000007779	RP: CTGTCCAAGACATCTACCATGATGA
	ENSMMUT00000007773	TP: AAGTTTTAGAGACAGTCTTC
*Pdk4*	ENSMMUT00000030108	FP: TGCACCAACGCCTGTGAT
		RP: GCAAGCCGTAACCAAAACCA
		TP:TTCCCGGAATGCTCCT

Sequences of primers; forward (FP), reverse (RP), Taqman probe (TP) specific to gene transcripts analyzed.

### Glycogen analysis

Glycogen content in the ventricle (LV, RV) of the LG group fetuses (control, *n = 5* and BPA, *n = 4*) was assessed using a modified colorimetric method [35]. Pre-weighed ground tissue (5–20 mg) in 0.3 ml of 30% KOH was boiled for 30 minutes with mixing. Samples were cooled to room temperature, 0.1 ml of 1 M Na_2_SO_4_, and 0.8 ml of 100% ethanol was added and samples were boiled for 5 additional minutes. Samples were centrifuged at 15000 x g for 10 minutes at 4°C, supernatant was removed and pellets were washed twice in 0.2 ml of Millipore water and 0.4 ml of 100% ethanol. Pellets were resuspended in 0.2 ml of amyloglucosidase solution (0.3 mg/ml amyloglucosidase in 0.2 M NaOAc pH 4.8), incubated at 40°C for 3 hours. Subsequently, the reaction mix was diluted with 0.2 M NaoAc; pH 4.8. Quantification of glucose, derived using hexokinase and glucose 6 – phosphate dehydrogenase from tissue glycogen, was measured at 340 nm using Evolution 300 UV -Vis spectrophotometer (Thermo scientific, Waltham, MA, USA).

### Microarray data analysis

NimbleScan software (NimbleGen. Madison, WI) was used to align a chip-specific grid to control features and extract raw intensity data for each probe and each array. Raw intensity data were then read into the R statistical computing environment and checked for quality (R Development Core Team [2010]: A language and environment for statistical computing R Foundation for Statistical Computing, Vienna, Austria ISBN 3-900051-07-0 URL http://www.R-project.org). Further, chip intensity distributions, boxplots and hierarchical clusters were compared and evaluated for any unusual global patterns. Within each tissue, each array was background corrected and normalized using the quantile normalization procedure and each probeset was summarized using the median polish procedure as described by the robust multichip average (RMA) procedure. Probesets with low levels of expression variation across all samples (IQR<0.5) were removed from further analysis, reducing the overall number of statistical tests to be performed. Differential expression was assessed using a linear model with an empirical Bayesian adjustment to the variances and comparisons of interest were extracted using contrasts *(n = 3)*. Genes that differ by an arbitrary differential expression filter criteria (DEFC) of log_2_ fold change (LFC) = ±1 at an unadjusted *p*≤0.01, were considered significantly altered in expression. Both the raw and RMA normalized microarray data were deposited in the Gene Expression Omnibus (GEO) database (accession number. GSE53393).

### Quantitative real-time PCR and glycogen data analysis

Taqman based gene expression analysis using qRT-PCR was performed as described previously [34], [36]. Briefly, delta Ct (ΔCt) values for each gene and all samples were obtained through subtracting the matched *18* *s rRNA* values from the averaged threshold cycle (Ct) values of candidate genes. Delta Ct values (ΔCt) served as the response variables (control, *n = 5* and BPA, *n = 4*) for statistical comparisons. Differential average response of each gene for BPA exposed over control (ΔΔCt) is reported as mean log_2_ fold change (LFC) ± SEM, and represented graphically using Sigma plot 8.0 software (SPSS Inc., Chicago, IL, USA). Tissue glycogen content was expressed as µg/mg wet tissue weight. Data were analyzed using SAS software (Statistical Analysis Software, Cary, NC, USA). A two sample t-test was performed to identify mean significant differences in the qRT-PCR measured gene expression and the glycogen content between the BPA exposed fetuses and the matching control group. A two-tailed *p*≤0.05 was considered statistically significant.

## Results

### Gene expression changes observed using whole genome DNA microarray

**Figure 1:**
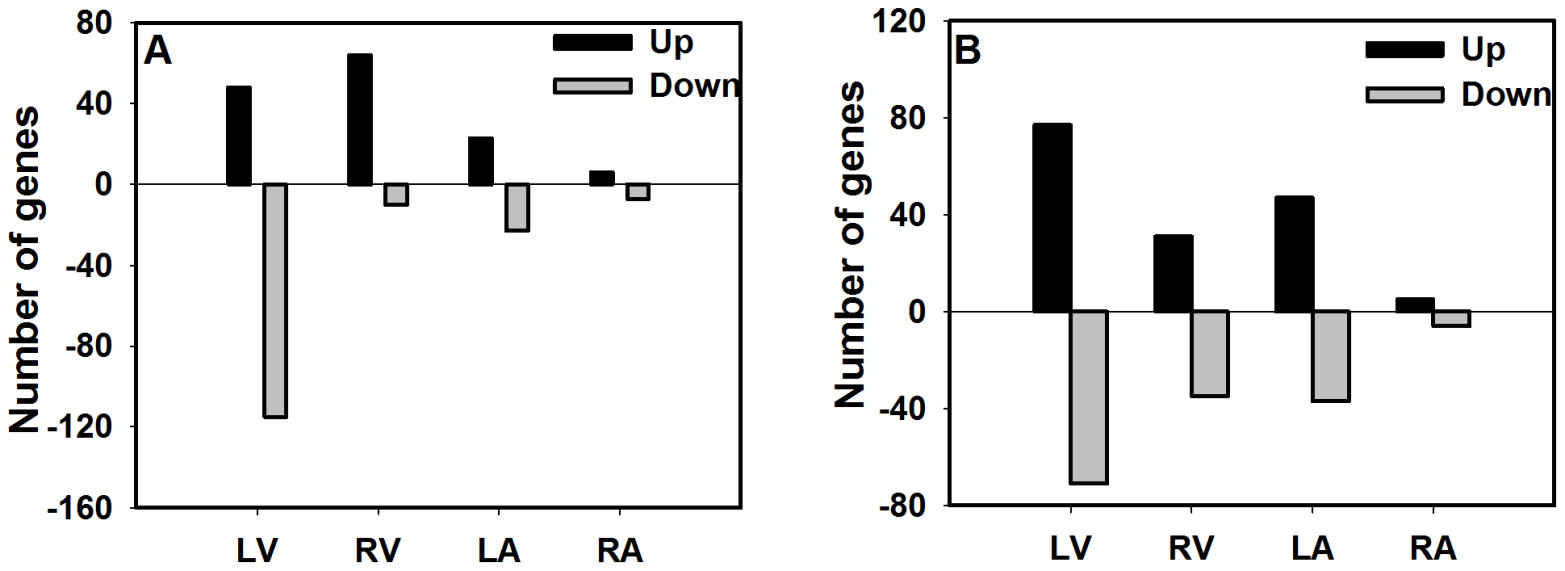
Total number of significantly altered genes observed using microarray global transcriptome expression analysis. Whole transcriptome analysis was performed on the left ventricle (LV), right ventricle (RV), left atrium (LA) and the right atrium (RA) of rhesus monkey (Macaca mulatta) fetuses that were exposed maternally to a 400 µg/kg body weight, Bisphenol A (BPA), relative to matched control fetuses, either during, (A) early gestation, EG (days; 50–100±2) or, (B) late gestation, LG (days 100±2–term). Genes that changed by greater than ±1 log_2_ fold change (1 LFC = 2 fold change) at an unadjusted p≤0.01 were considered significant and differentially expressed. Bars represent the number of upregulated (positive y-axis) or downregulated (negative y-axis) genes in each of the tissues.

Microarray analysis of cardiac tissues (LV, RV, LA & RA) indicated a differential mRNA expression profile in the EG and the LG maternal BPA exposed fetuses compared to that of matching controls. Genes that changed by ≥2 fold (LFC = ±1) at p≤0.01 (unadjusted *p-value*) were considered significantly altered by maternal BPA exposure. This resulted in lists of up- or down-regulated genes in cardiac tissues analyzed after EG (Fig. 1A; Table S1–S4) or LG (Fig. 1B; Table S5–S8) maternal BPA treatment. Differentially expressed genes included both protein coding genes and a variety of non-protein coding RNAs; micro RNAs (miRNAs), small nucleolar RNAs and other RNAs. Manual screening of the differentially expressed gene lists identified two important protein coding genes that changed significantly; *Myh6* and *Adam12*, and both these genes were recognized for their role in cardiac pathophysiologies. Our results show that *Myh6* is significantly down-regulated in the fetal LV (LFC = −5.385, ≈42 fold), but not in the RV or the atria, after LG maternal BPA exposure, or in any tissue type after EG maternal BPA exposure, compared to the matched controls (Table 2–3). On the other hand, *Adam12* is significantly up-regulated in the LV (LFC = 1.678, ≈3.2 fold) and the RV (LFC = 1.213, ≈2.3 fold) of the LG, but not the atria of the LG, or in any tissue of the EG, maternal BPA exposed fetuses compared to matched controls (Table 2–3).

**Table 2:** Differentially expressed genes observed using microarray on rhesus fetal heart exposed maternally to BPA or vehicle during early gestation, EG (days; 50–100±2).

ENSEMBL_ID	Gene symbol	EG - log_2_ fold change			
		LV	RV	LA	RA
ENSMMUG00000014754	*Myh6*	−0.204	2.619	ND	ND
ENSMMUG00000009661	*Adam12*	0.09	−0.115	−0.225	0.192
ENSMMUG00000000067	*Thrb*	0.845	−0.441	ND	0.536
ENSMMUG00000005521	*Gyg1*	ND	−0.177	ND	ND
ENSMMUG00000021389	*Pdk4*	0.498	0.188	0.016	−0.263
ENSMMUG00000026923	*mml-mir-205*	−0.183	ND	−0.385	0.403
ENSMMUG00000026877	*mml-mir-224*	0.581	ND	−0.459	−0.069

ND - below differential expression filter criteria.

**Table 3:** Differentially expressed genes observed using microarray on rhesus fetal heart exposed maternally to BPA or vehicle during early gestation, LG (days; 100±2–term).

ENSEMBL_ID	Gene symbol	LG - log_2_ fold change			
		LV	RV	LA	RA
ENSMMUG00000014754	*Myh6*	−5.385	−0.187	ND	ND
ENSMMUG00000009661	*Adam12*	1.678	1.213	0.091	0.555
ENSMMUG00000000067	*Thrb*	1.185	−0.865	ND	−0.096
ENSMMUG00000005521	*Gyg1*	ND	−0.143	ND	ND
ENSMMUG00000021389	*Pdk4*	0.346	0.171	0.22	0.322
ENSMMUG00000026923	*mml-mir-205*	2.583	ND	−0.909	0.109
ENSMMUG00000026877	*mml-mir-224*	4.093	ND	0.275	0.161

ND - below differential expression filter criteria.

Thyroid hormone status and glycolytic flux are important determinants of *Myh6* expression (Gupta 2007). Accordingly, thyroid hormone receptors play an important role in the *Myh6* expression, and thyroid hormone receptor beta (Q8SPN6_MACMU - *Thrb*) is up-regulated in the LV of the LG, BPA treated fetuses *vs.* matched controls (Table 3). However, this did not meet our differential expression filter criteria as the unadjusted *p = 0.055*. Also, preliminary studies in our lab indicate that maternal BPA exposure may interfere with the glycogen metabolism in mice fetal heart tissue as measured through altered pyruvate dehydrogenase kinase isozyme 4 (*Pdk4*) and glycogenin (*Gyg1*) mRNA expression (data not shown). Microarray analyses revealed no significant differences in the mRNA expression levels of either *Pdk4* or *Gyg1* between the maternal BPA exposed *vs.* matched control fetuses (Table 2–3).

We also observed induction of two miRNAs; *miR-205* (LFC = 2.583, ≈6 fold) and *miR-224* (LFC = 4.093, ≈17 fold) in LV of the LG, maternal BPA exposed fetuses compared to that of the matching controls (Table 2–3). Collectively, microarray results indicate that maternal BPA exposure alters the transcriptional profile in rhesus fetal heart pertaining to cardiac remodeling events that have been linked to cardiovascular pathophysiologies.

### Quantitative real-time PCR analysis

**Figure 2:**
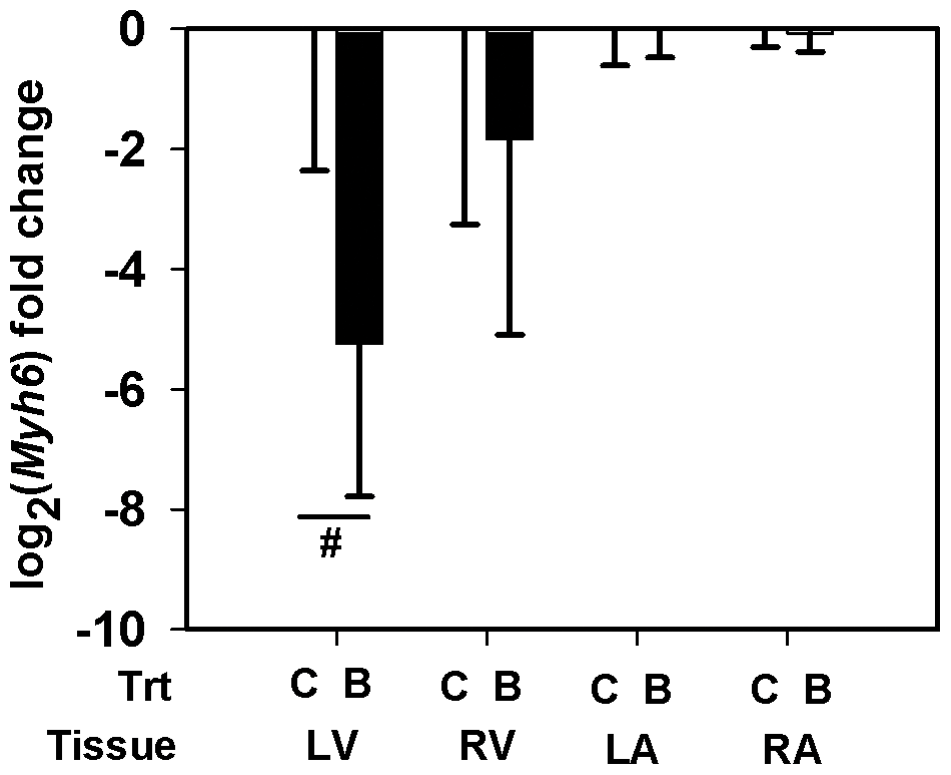
Altered fast myosin heavy chain expression. Real-time PCR quantification of myosin heavy chain 6, cardiac isoform alpha (Myh6) expression in the left ventricle (LV), right ventricle (RV), left atrium (LA) and the right atrium (RA) of rhesus monkey (Macaca mulatta) fetuses at birth. Fetuses were exposed maternally to a 400 µg/Kg. body weight of BPA dose (B) or 150 µL ethanol (C) during late gestation (LG, days 100±2–term). Data were analyzed by tissue and bars represent the mean ± S.E.M. of the log_2_ (fold change). A two sample t-test was performed to identify significant BPA effects and a two-tailed p≤0.05 was considered statistically significant. Marginal significance between the groups was indicated with a ‘#’ symbol.

**Figure 3:**
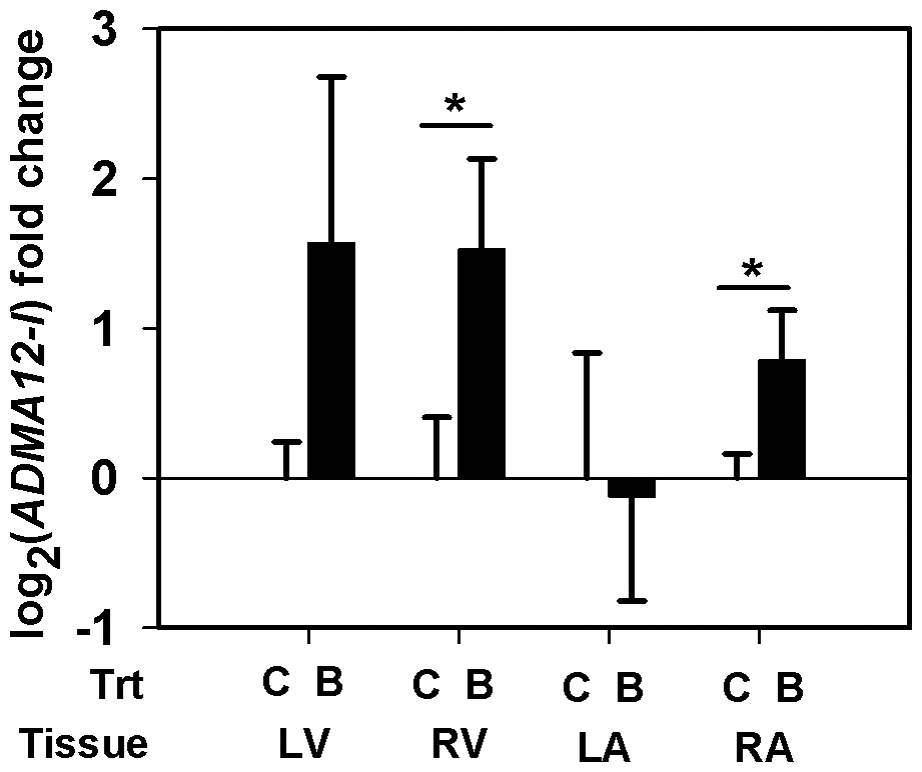
Expression changes in Adam12-l mRNA. Real-time PCR quantification of the long isoform of A Disintegrin And Metalloprotease (Adam12-l) expression in the left ventricle (LV), right ventricle (RV), left atrium (LA) and the right atrium (RA) of rhesus monkey (Macaca mulatta) fetuses at birth. Fetuses were exposed maternally to a 400 µg/Kg. body weight of BPA dose (B) or 150 µL ethanol (C) during late gestation (LG, days 100±2–term). Data were analyzed by tissue and bars represent the mean ± S.E.M. of the log_2_ (fold change). A two sample t-test was performed to identify significant BPA effects and a two-tailed p≤0.05 was considered statistically significant. Asterisk (*) indicate significantly different groups.

**Figure 4:**
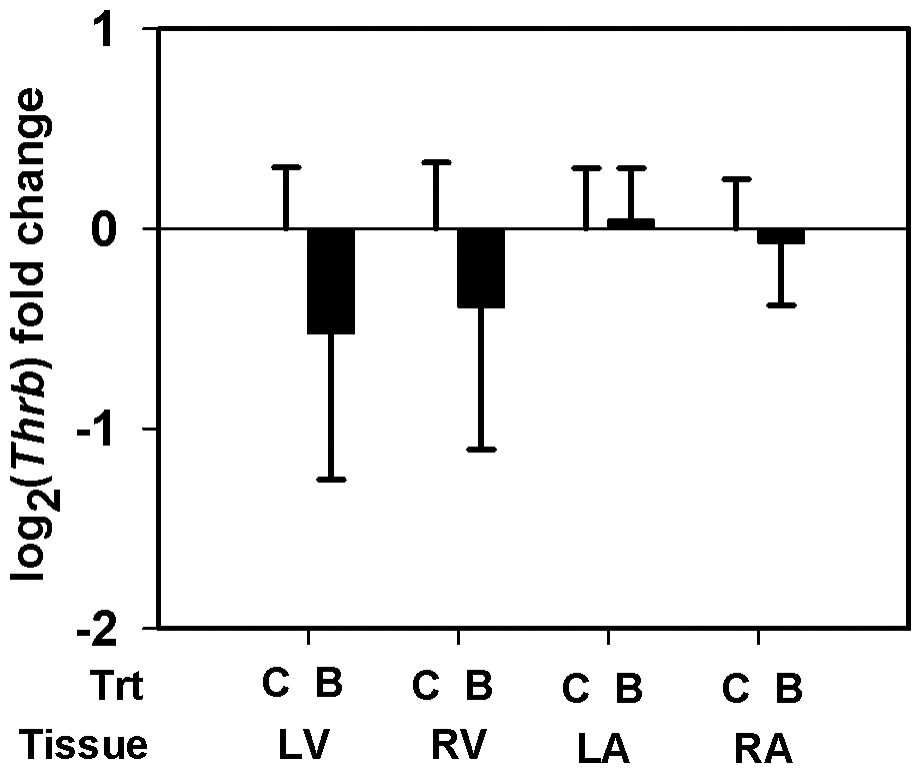
Real-time PCR quantification of thyroid hormone receptor beta (Thrb) expression. Transcript levels of Thrb in the left ventricle (LV), right ventricle (RV), left atrium (LA) and the right atrium (RA) of rhesus monkey (Macaca mulatta) fetuses at birth. Fetuses were exposed maternally to a 400 µg/Kg. body weight of BPA dose (B) or 150 µL ethanol (C) during late gestation (LG, days 100±2–term). Data were analyzed by tissue and bars represent the mean ± S.E.M. of the log_2_ (fold change). A two sample t-test was performed to identify significant BPA effects and a two-tailed p≤0.05 was considered statistically significant.

**Figure 5:**
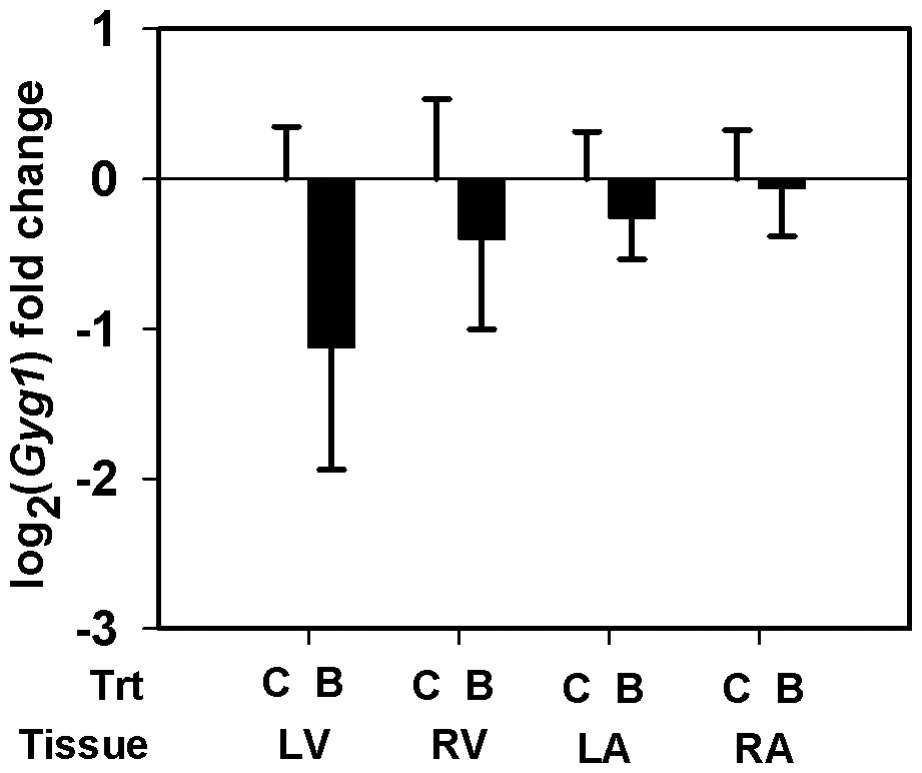
Real-time PCR quantification of glycogenin (Gyg1) expression. Expression of Gyg1 mRNA in the left ventricle (LV), right ventricle (RV), left atrium (LA) and the right atrium (RA) of rhesus monkey (Macaca mulatta) fetuses at birth was measured. Fetuses were exposed maternally to a 400 µg/Kg. body weight of BPA dose (B) or 150 µL ethanol (C) during late gestation (LG, days 100±2–term). Data were analyzed by tissue and bars represent the mean ± S.E.M. of the log_2_ (fold change). A two sample t-test was performed to identify significant BPA effects and a two-tailed p≤0.05 was considered statistically significant.

**Figure 6:**
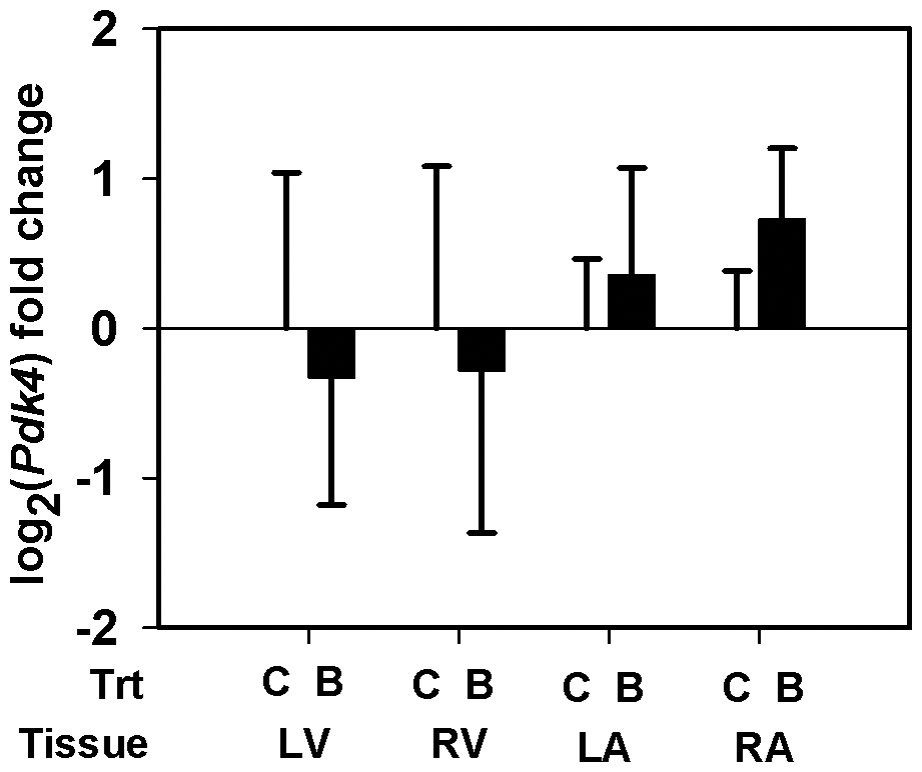
Pyruvate dehydrogenase kinase isozyme 4 (Pdk4) mRNA expression. Real-time PCR quantification of Pdk4 gene was performed on the left ventricle (LV), right ventricle (RV), left atrium (LA) and the right atrium (RA) samples of rhesus monkey (Macaca mulatta) fetuses at birth. Fetuses were exposed maternally to a 400 µg/Kg. body weight of BPA dose (B) or 150 µL ethanol (C) during late gestation (LG, days 100±2–term). Data were analyzed by tissue and bars represent the mean ± S.E.M. of the log_2_ (fold change). A two sample t-test was performed to identify significant BPA effects and a two-tailed p≤0.05 was considered statistically significant.

Using qRT-PCR, we quantified the transcript abundance of selected genes in all the heart tissue types from the LG maternal BPA exposed fetuses (*n = 4*) and the matching controls (*n = 5*) (Table 1). Myosin isoform *Myh6* specific mRNA expression was measured. Two isoforms of *Adam12*, the membrane anchored long isoform (*Adam12-l*) and the secreted short isoform (*Adam12-s*), arise through alternative splicing of *Adam12* transcript. Using a specific primer probe set for *Adam12-l* (Table 1), we measured and report the transcript abundance of *Adam12-l* isoform. Similar results were obtained when we used another primer-probe set that simultaneously detects both isoforms (data not shown) indicating the predominant expression of the long isoform in these tissues. Although the *Thrb*, *Gyg1* and *Pdk4* genes did not pass our filtering or significance criteria in the microarray study (Table 2–3), we elected to quantify mRNA expression for these genes using the more sensitive qRT-PCR. Expression of *Myh6* mRNA was down-regulated by ≈38 fold (LFC = − 5.243±2.544, *p* = 0.077) in the LV of maternal BPA exposed fetuses compared to matching controls (Fig. 2). While the difference is marginally significant, we believe this observation is important to report since the variability is largely caused by one fetus in each group. Also, *Adam12-l* expression increased similarly in both ventricles, reaching significance in the RV (LFC = 1. 528±0. 6039, ≈2.9 fold, *p* = 0. 031) and trending in the LV (LFC = 1.57±1. 1087, ≈3 fold, *p* = 0.154), of the BPA exposure group *vs.* matched control (Fig. 3). Increased *Adam12-l* expression was also observed in the RA of the BPA treatment group compared to controls (LFC = 0.788±0.3317, ≈1.73 fold, *p* = 0.035) (Fig. 3), perhaps not surprising considering this value in the microarray experiment for the RA was ≈1 fold (LFC = 0.555). No significant changes in expression of *Thrb* (Fig. 4) *Gyg1* (Fig. 5) and *Pdk4* (Fig. 6) were observed between the BPA exposed and control groups. Collectively, these qRT-PCR results largely confirm the microarray expression changes.

### Tissue glycogen content

**Figure 7:**
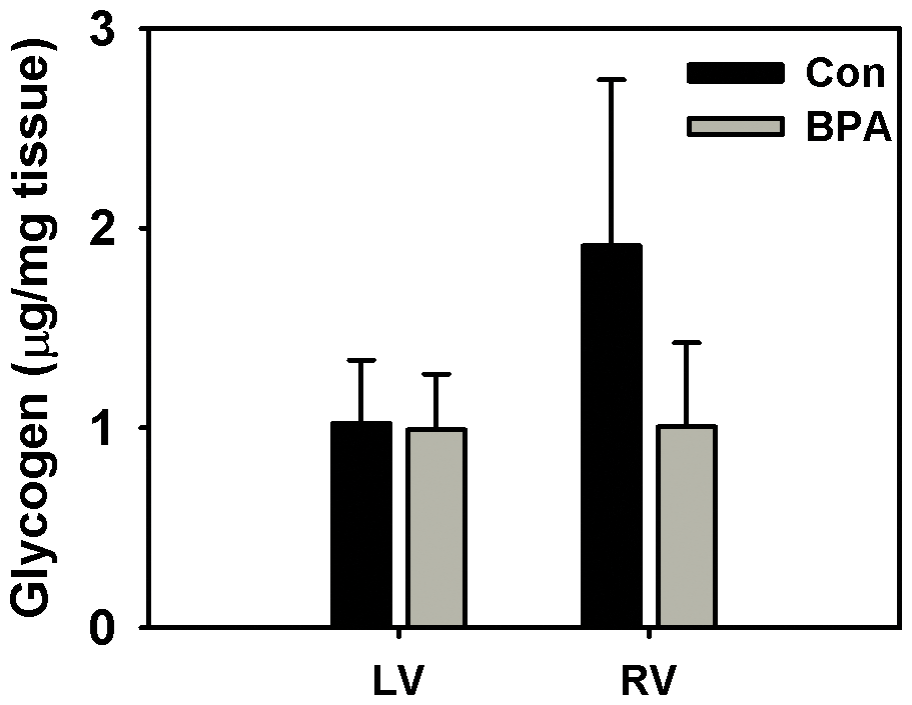
Tissue glycogen estimation. Ventricular glycogen content was assessed using a calorimetric method on the left ventricle (LV) and the right ventricle (RV) of rhesus monkey (Macaca mulatta) fetuses at birth. Fetuses were exposed maternally to a 400 µg/Kg. body weight of BPA dose (B) or 150 µL ethanol (C) during late gestation (LG, days 100–term). Data were analyzed by tissue and bars represent the mean ± S.E.M., of the tissue glycogen content measured in µg for every milligram tissue. A two sample t-test at a two-tailed p≤0.05 was considered statistically significant.

Glycogen is an important source of glucose and hence energy in the newborn fetus, therefore we measured the tissue glycogen content towards gaining insight into the mechanism of *Myh6* down-regulation at the metabolite level. However, ventricular muscle glycogen, in the LG maternal BPA exposed *vs.* matched control fetuses was not significantly different (Fig. 7).

## Discussion

We surveyed the broad-spectrum effects of maternal BPA exposure on female fetal cardiac gene expression profiles in a non-human primate using microarray and qRT-PCR analyses. We report evidence of BPA-induced gene expression changes in the primate fetal cardiovascular tissue indicating potential altered cardiac development. This study provides insights into the potential consequences of BPA exposure on human development and long-term health.

Maternal BPA exposure during LG causes a down-regulation of fast myosin heavy chain isoform of mammalian myocardium (*Myh6*) mRNA expression in the LV of rhesus fetal heart. *Myh6* gene products' contribution to total myocardial myosin, vary by tissue (ventricle *vs.* atrium), species (rodents *vs.* humans) and developmental stage (fetal *vs.* adult) [37]–[39]. While less abundant in the fetal ventricles, *Myh6* induction normally occurs just prior to birth and predominates in the rodent ventricles (>95%) while representing approximately 7% of the total myosin protein in human adult left ventricles [38]–[40]. Importantly, down-regulation of *Myh6* to nearly undetectable levels in the failing adult human left ventricles has been noted [38], [40], indicating that this myosin isoform influences ventricular myocardial fitness. Research on breast cancer cell lines indicates an inhibitory effect of 17β-estradiol, BPA and other known endocrine disruptor compounds; genistein and polychlorinated biphenyl congener 54 (PCB54), on the *Myh6* transcript levels [41]. Although, the functional significance of *Myh6* in breast cancer cells is unknown, these studies do provide a model of how BPA may down-regulate *Myh6*. Given that neonatal cardiomyocytes express functional estrogen receptors [23] it is plausible that hormone mediated pathways drive the BPA induced inhibition of *Myh6* expression observed in our study.

Thyroid hormone status is an important determinant of myosin isoform composition in the cardiac ventricles [42]. Initiation of thyroid hormone receptor alpha binding of T_3_ at birth initiates the onset of postnatal cardiac phenotype [43] and myosin heavy chain isoforms in postnatal ventricles are direct transcriptional targets of thyroid hormone as indicated by reduced *Myh6* expression in hypothyroid adult ventricular myocardium [44]–[47]. Research in rodent cells revealed that BPA acts as a thyroid hormone antagonist and represses thyroid hormone transcriptional target genes by non-competitively binding to the thyroid hormone receptor, and recruiting a co-repressor, N-CoR [6], [7]. Our result supports this mechanism of action and indicates that BPA inhibits the induction of *Myh6* and may interfere with the transformation of the fetal heart into postnatal heart. Another non-exclusive mechanism of *Myh6* down-regulation may be the repression of thyroid hormone receptor expression [48]. However, we did not observe any significant differences in the mRNA expression of these receptors in response to BPA exposure. A recent report showed that *miR-205* silences TRAP220/MED1, a co-activator of several nuclear hormone receptors including thyroid hormone receptor, in hypoxic human placental trophoblasts [49]. Induction of *miR-205* in our study is particularly important as TRAP220 is indispensable for embryonic development and homozygous TRAP220 knockout mice experience placental insufficiency and defective heart development, consistent with heart failure, and suffer early embryonic mortality [50], [51]. *In vitro* studies conducted on embryonic fibroblasts from these TRAP220 knockout mice indicated perturbed thyroid hormone signaling. The BPA mediated induction of *miR-205* and *miR-224* in fetal cardiac tissue is compelling as these are known to regulate tissue remodeling [49], [52].

Maternal BPA exposure during LG up-regulates *Adam12-l* expression in the LV (microarray), RV and RA (microarray and qRT-PCR) of rhesus monkey fetuses. *Adam12* is a member of the ADAMs multi-gene family that is expressed during early embryonic and fetal stages of development and in the adult heart [53], [54] with demonstrated roles in the integration of cell-to-cell and cell-to-extracellular matrix interaction, as well as growth factor signaling [55]. *Adam12* has been implicated in the development of cardiac hypertrophy [56] and elevated expression of *Adam12* is observed in human and rodent cardiac tissue during hypertrophic obstructive cardiomyopathy [53] and agonist-induced cardiac hypertrophy [57], [58]. Although the physiological pathway associated with up-regulation of *Adam12-l* is not clear, we speculate that the transforming growth factor beta (TGFβ) pathway may be involved. Research conducted using hepatic stellate cells [59], [60], fibroblasts and mammary epithelial cells [61], reported that TGFβ, isoform 1 (TGFβ1) is a transcriptional activator of both the long and the short isoforms of *Adam12* that reciprocally promotes TGFβ/smad signaling by enhancing the TGFβ type II receptor internalization [62]. Further, BPA up-regulates the expression of other *TGFβ* isoforms, *TGFβ2* and *TGFβ3* transcripts but not *TGFβ1*, in neural tissue [63] and endometrial cancer cells [64], respectively. Additionally, up-regulation of *miR-224* observed in our study further supports this interpretation as TGFβ1 was shown to activate *miR-224* transcription in mouse granulosa cells [52]. Lack of any significantly increased *TGFβ* mRNA expression in our microarray analysis (data not shown) is however not surprising as temporal differences may exist in the expression of both the *Adam12-l* and *TGFβ* transcripts.

Our microarray analysis shows that BPA caused largely distinct gene expression changes in different cardiac quadrants and at both the developmental stages. Indeed, different quadrants of the mammalian heart (LV, RV, LA & RA) are divergent in structure, contractile function and metabolism as confirmed by compartment specific myocardial gene expression profiles [65]–[67]. Moreover, cardiac remodeling events that underlie important cardiovascular pathophysiologies vary in accordance with the specific heart compartments [67]–[69]. Hence, the influence of BPA on different anatomical subdivisions of the heart is presumed to be variable. Additionally, we observed larger ‘within group’ variability in gene expression, which may signify the genetic heterogeneity in the sample groups studied. While gene expression studies would benefit from a larger sample size, nonetheless our findings offer the first evidence of the potential of the maternal BPA exposure to alter cardiac development and health in non-human primate rhesus monkey.

In conclusion, our data indicate that chronic exposure to maternally delivered BPA significantly alters the transcript expression profiles of several coding and non-coding genes in rhesus monkey fetal heart tissue. The results of this study are important for multiple reasons. First, the observed changes of *Myh6* and *Adam12-l* mRNA expression are consistent with events that underlie cardiac remodeling during cardiovascular pathophysiologies such as cardiac hypertrophy [38], [40], [56]. Secondly, similarities in the metabolism of rhesus monkey and humans make these observations relevant to the latter, and may provide physiological insights into the recently reported correlations between urinary BPA concentration and the incidence of cardiovascular disease [20], [21]. Finally, our results emphasize the impact of physiologically relevant BPA exposure as the average maternal serum unconjugated BPA level in our study was 0.68 ng/ml [29], which is closer to the levels reported in human maternal serum during gestation [70], [71]. The results of this study conflict with recent reports that suggest that BPA poses no human health risk in either adults or newborns [72]. This research adds to the evidence suggesting that the rising incidence of cardiovascular and metabolic diseases in the human population may be in part a reflection of our lifetime dietary and environmental exposure to chemicals such as BPA.

## Supporting Information

Table S1 **List of gene transcripts that changed by ≥2 fold (log_2_ fold change (LFC) = ±1), at** ***p*** **≤0.01 (unadjusted), in the left ventricle (LV) of the early gestation (EG), maternally BPA exposed** ***vs.*** **matched control, fetuses.**
(PDF)

Table S2 **List of gene transcripts that changed by ≥2 fold (log_2_ fold change (LFC) = ±1) at** ***p*** **≤0.01 (unadjusted), in the right ventricle (RV) of the early gestation (EG), maternally BPA exposed** ***vs.*** **matched control, fetuses.**
(PDF)

Table S3 **List of gene transcripts that changed by ≥2 fold (log_2_ fold change (LFC) = ±1), at** ***p*** **≤0.01 (unadjusted), in the left atrium (LA) of the early gestation (EG), maternally BPA exposed** ***vs.*** **matched control, fetuses.**
(PDF)

Table S4 **List of gene transcripts that changed by ≥2 fold (log_2_ fold change (LFC) = ±1), at** ***p*** **≤0.01 (unadjusted), in the right atrium (RA) of the early gestation (EG), maternally BPA exposed** ***vs.*** **matched control, fetuses.**
(PDF)

Table S5 **List of gene transcripts that changed by ≥2 fold (log_2_ fold change (LFC) = ±1), at** ***p*** **≤0.01 (unadjusted), in the left ventricle (LV) of the late gestation (LG), maternally BPA exposed** ***vs.*** **matched control, fetuses.**
(PDF)

Table S6 **List of gene transcripts that changed by ≥2 fold (log_2_ fold change (LFC) = ±1), at** ***p*** **≤0.01 (unadjusted), in the right ventricle (RV) of the late gestation (LG), maternally BPA exposed** ***vs.*** **matched control, fetuses.**
(PDF)

Table S7 **List of gene transcripts that changed by ≥2 fold (log_2_ fold change (LFC) = ±1), at** ***p*** **≤0.01 (unadjusted), in the left atrium (LA) of the late gestation (LG), maternally BPA exposed** ***vs.*** **matched control, fetuses.**
(PDF)

Table S8 **List of gene transcripts that changed by ≥2 fold (log_2_ fold change (LFC) = ±1), at** ***p*** **≤0.01 (unadjusted), in the right atrium (RA) of the late gestation (LG), maternally BPA exposed** ***vs.*** **matched control, fetuses.**
(PDF)
