# Supplementary material for: Maternal Bisphenol A Exposure Impacts the Fetal Heart Transcriptome
Source: PLoS One. 2014 Feb 25;9(2):e89096. doi: 10.1371/journal.pone.0089096 (PMC3934879; doi:10.1371/journal.pone.0089096)
Supplement: Table S1 — List of gene transcripts that changed by ≥2 fold (log2 fold change (LFC) = ±1), at p ≤0.01 (unadjusted), in the left ventricle (LV) of the early gestation (EG), maternally BPA exposed vs. matched control, fetuses. (PDF) [file pone.0089096.s001.pdf]

Table S1. List of gene transcripts that changed by ≥ 2 fold (log_2 fold change (LFC) = ± 1), at *p* ≤ 0.01 (unadjusted), in the left ventricle (LV) of the early gestation (EG), maternally BPA exposed *vs.* matched control, fetuses.

SEQ_ID	Gene description	log_2 fold change[a]	*p* value
ENSMMUT00000049707	mml-mir-563	5.323	0.001
ENSMMUT00000036245	Y RNA	4.669	0.006
ENSMMUT00000036754	mml-mir-194-1	4.057	0.004
ENSMMUT00000036732	mml-mir-135a-1	3.916	0.004
ENSMMUT00000036236	Y RNA	3.877	0.01
ENSMMUT00000050262	5S ribosomal RNA	3.441	0.009
ENSMMUT00000033723	Small nucleolar RNA SNORA2/SNORA34 family	3.203	0.002
ENSMMUT00000035173	Small nucleolar RNA SNORA41	3.044	0.006
ENSMMUT00000035699	Small nucleolar RNA SNORD113/SNORD114 family	2.886	0.001
ENSMMUT00000016931	novel protein_coding	2.824	0.002
ENSMMUT00000005699	Zinc finger SWIM domain-containing protein 2	2.814	0.004
ENSMMUT00000048998	Small nucleolar RNA SNORA57	2.754	0.006
ENSMMUT00000050231	Eukaryotic type signal recognition particle RNA	2.727	0.006
ENSMMUT00000018990	Placenta-specific 1-like protein Precursor	2.661	0
ENSMMUT00000027509	Olfactory receptor 8B4	2.661	0.004
ENSMMUT00000004910	Novel protein_coding	2.627	0.001
ENSMMUT00000050458	7SK RNA	2.547	0.005
ENSMMUT00000034332	U1 spliceosomal RNA	2.514	0.008
ENSMMUT00000003381	Interferon alpha-inducible protein 27-like protein 1	2.451	0.008
ENSMMUT00000010137	Synaptic vesicle glycoprotein 2A	2.321	0.007
ENSMMUT00000034245	5S ribosomal RNA	2.248	0.001
ENSMMUT00000036839	Novel miRNA	2.169	0.006
ENSMMUT00000007788	Serine/threonine-protein kinase MAK	2.129	0.003
ENSMMUT00000005248	Family 13, member C1-like protein	2.127	0.01
ENSMMUT00000034795	U1 spliceosomal RNA	2.09	0.002
ENSMMUT00000014825	tumor necrosis factor	1.923	0.007
ENSMMUT00000007275	Olfactory receptor 9Q2	1.88	0.005
ENSMMUT00000002411	Stonin-1	1.81	0.003
ENSMMUT00000010742	Desmocollin-3 Precursor	1.764	0.003
ENSMMUT00000011791	Rho-related GTP-binding protein Rho6 Precursor	1.728	0.007
ENSMMUT00000008899	Synaptonemal complex central element protein 1	1.726	0.006
ENSMMUT00000021372	interleukin-18	1.716	0.006
ENSMMUT00000010384	Novel protein_coding	1.71	0.008
ENSMMUT00000006441	HCG1777656Putative uncharacterized proteinPutative uncharacterized protein GSTTP1	1.709	0.007
ENSMMUT00000050527	Eukaryotic type signal recognition particle RNA	1.682	0.008
ENSMMUT00000014919	HORMA domain-containing protein 1	1.667	0.009
ENSMMUT00000018988	Gastric intrinsic factor Precursor	1.66	0.007
ENSMMUT00000042406	Prominin-1 Precursor	1.646	0.005
ENSMMUT00000016453	RING finger protein 32	1.611	0.006
ENSMMUT00000022193	Kelch-like protein 17	1.592	0.008
ENSMMUT00000043977	known protein_coding	1.544	0.005
ENSMMUT00000040878	Serine protease inhibitor Kazal-type 5 Precursor	1.513	0.003

ENSMMUT00000034586	U1 spliceosomal RNA	1.485	0.006
ENSMMUT00000005790	Kelch repeat and BTB domain-containing protein 8	1.395	0.007
ENSMMUT00000042488	Protein PMS2CL	1.282	0.008
ENSMMUT00000020666	Novel protein_coding	1.233	0.007
ENSMMUT00000031838	Novel protein_coding	1.185	0.009
ENSMMUT00000003676	Sodium-dependent phosphate transport protein 1	1.164	0.008
ENSMMUT00000049939	Eukaryotic type signal recognition particle RNA	-5.681	0.002
ENSMMUT00000036475	U6 spliceosomal RNA	-5.556	0.005
ENSMMUT00000048686	mml-mir-652	-4.778	0.001
ENSMMUT00000034471	U6 spliceosomal RNA	-4.73	0.009
ENSMMUT00000049501	mml-mir-557	-4.702	0.01
ENSMMUT00000036363	Y RNA	-4.364	0.007
ENSMMUT00000037154	U6 spliceosomal RNA	-4.228	0.002
ENSMMUT00000042181	novel protein_coding	-4.203	0
ENSMMUT00000049678	U6atac minor spliceosomal RNA	-4.19	0.003
ENSMMUT00000050795	U6 spliceosomal RNA	-3.993	0.008
ENSMMUT00000037441	U6 spliceosomal RNA	-3.881	0.009
ENSMMUT00000035286	Small nucleolar RNA SNORD74	-3.76	0.002
ENSMMUT00000034022	Y RNA	-3.733	0.008
ENSMMUT00000036466	U6 spliceosomal RNA	-3.694	0.006
ENSMMUT00000048820	U6 spliceosomal RNA	-3.593	0.002
ENSMMUT00000034578	Small nucleolar RNA U3	-3.47	0.004
ENSMMUT00000006829	Protein S100-A7-like 2	-3.441	0.005
ENSMMUT00000049190	Novel miRNA	-3.313	0.007
ENSMMUT00000033879	U5 spliceosomal RNA	-3.206	0.001
ENSMMUT00000034186	Y RNA	-3.173	0.003
ENSMMUT00000034986	Y RNA	-3.104	0.005
ENSMMUT00000050093	Eukaryotic type signal recognition particle RNA	-3.053	0.001
ENSMMUT00000050336	Eukaryotic type signal recognition particle RNA	-3.041	0.003
ENSMMUT00000049721	Small Nucleolar RNA SNORD111	-2.996	0.002
ENSMMUT00000031762	Novel protein_coding	-2.981	0.006
ENSMMUT00000026546	Follicle stimulating hormone beta polypeptide	-2.897	0.004
ENSMMUT00000034178	U6 spliceosomal RNA	-2.79	0.002
ENSMMUT00000020628	Novel protein_coding	-2.783	0.005
ENSMMUT00000035806	Y RNA	-2.768	0.005
ENSMMUT00000035283	U6 spliceosomal RNA	-2.744	0.009
ENSMMUT00000010349	Arrestin domain-containing protein 2	-2.739	0.001
ENSMMUT00000036368	5S ribosomal RNA	-2.723	0.003
ENSMMUT00000001955	Gap junction alpha-8 protein	-2.72	0.009
ENSMMUT00000050062	Small subunit ribosomal RNA, 5' domain	-2.695	0.004
ENSMMUT00000030663	RasGAP-activating-like protein 1	-2.685	0.007
ENSMMUT00000007224	Tumor necrosis factor ligand superfamily member 8	-2.668	0.003
ENSMMUT00000051159	5S ribosomal RNA	-2.657	0.001
ENSMMUT00000021114	Histamine H4 receptor	-2.64	0.001
ENSMMUT00000034667	U4 spliceosomal RNA	-2.597	0.007
ENSMMUT00000029172	Olfactory receptor 8B12	-2.576	0.002
ENSMMUT00000020584	Olfactory receptor	-2.573	0.004

ENSMMUT00000018313	Hyaluronan synthase 1	-2.558	0.007
ENSMMUT00000049889	7SK RNA	-2.551	0.008
ENSMMUT00000003938	Hepatocyte nuclear factor 4-gamma	-2.538	0.005
ENSMMUT00000033980	U6 spliceosomal RNA	-2.53	0.001
ENSMMUT00000033787	Small nucleolar RNA SNORA38	-2.525	0.009
ENSMMUT00000002882	Interstitial collagenase Precursor	-2.51	0.003
ENSMMUT00000023620	Paired box protein Pax-6	-2.463	0.003
ENSMMUT00000038016	5S ribosomal RNA	-2.435	0.004
ENSMMUT00000005486	Sal-like protein 4	-2.404	0.003
ENSMMUT00000050581	Eukaryotic type signal recognition particle RNA	-2.396	0.001
ENSMMUT00000021148	Vascular non-inflammatory molecule 3 Precursor	-2.367	0.008
ENSMMUT00000003335	Uncharacterized protein C4orf36	-2.323	0.007
ENSMMUT00000014656	Ankyrin repeat domain-containing protein 58	-2.309	0.004
ENSMMUT00000011226	Beta-microseminoprotein precursor	-2.304	0.007
ENSMMUT00000016182	Interleukin-16 precursor	-2.3	0.009
ENSMMUT00000007236	Neuroendocrine convertase 1 Precursor	-2.292	0.004
ENSMMUT00000037049	Y RNA	-2.276	0.001
ENSMMUT00000007864	Novel protein_coding	-2.269	0.005
ENSMMUT00000022594	Complement component 1, q subcomponent-like 1	-2.264	0.002
ENSMMUT00000015913	Uncharacterized protein C6orf223	-2.23	0
ENSMMUT00000025935	Novel protein_coding	-2.209	0.001
ENSMMUT00000001429	Solute carrier family 23 member 1	-2.2	0.007
ENSMMUT00000024779	Novel protein_coding	-2.184	0.006
ENSMMUT00000049611	Novel miRNA	-2.159	0.004
ENSMMUT00000044837	Growth hormone variant precursor	-2.11	0.004
ENSMMUT00000012142	Solute carrier family 28 member 3	-2.11	0.01
ENSMMUT00000022627	Nicotinic acetylcholine receptor subunit beta2	-2.104	0
ENSMMUT00000037794	U6 spliceosomal RNA	-2.086	0.001
ENSMMUT00000032007	WAP, kazal, immunoglobulin, kunitz & NTR domain-containing protein 2 Precursor	-2.048	0.006
ENSMMUT00000001481	Uncharacterized protein C8orf80	-2.032	0.003
ENSMMUT00000000391	Fibronectin type III and SPRY domain-containing protein 1	-2.027	0.008
ENSMMUT00000046073	Novel protein_coding	-2.025	0.004
ENSMMUT00000029331	C-type lectin domain family 4 member F	-2.014	0
ENSMMUT00000009882	Neutral ceramidase	-2.006	0.009
ENSMMUT00000000891	Uncharacterized NHL-repeat-containing protein C16orf11	-1.986	0.007
ENSMMUT00000042512	Novel protein_coding	-1.985	0.009
ENSMMUT00000015519	Protein FAM47A	-1.968	0.009
ENSMMUT00000049634	Novel miRNA	-1.965	0.008
ENSMMUT00000014197	Thioredoxin domain-containing protein 3	-1.958	0.004
ENSMMUT00000010814	Hemopexin Precursor	-1.954	0
ENSMMUT00000026787	Interferon omega-1 Precursor	-1.923	0.006
ENSMMUT00000026754	Novel protein_coding	-1.886	0.002
ENSMMUT00000014766	Mucin-5B Precursor	-1.872	0.009
ENSMMUT00000016195	Arylsulfatase I Precursor	-1.869	0.006
ENSMMUT00000026236	Polycystic kidney disease and receptor for egg jelly-related protein Precursor	-1.827	0.005
ENSMMUT00000025736	Retinoic acid receptor responder protein 3	-1.82	0.005

ENSMMUT00000015434	Novel protein_coding	-1.797	0.009
ENSMMUT00000000164	Novel protein_coding	-1.774	0.005
ENSMMUT00000020299	Ephrin type-A receptor 5 Precursor	-1.764	0.006
ENSMMUT00000025718	Secreted Ly-6/uPAR-related protein 1 Precursor	-1.744	0.007
ENSMMUT00000022802	Ankyrin repeat and LEM domain-containing protein 1	-1.713	0.002
ENSMMUT00000007726	Calcium-binding protein 4	-1.683	0.006
ENSMMUT00000001108	Olfactory receptor 2T6	-1.683	0.009
ENSMMUT00000040972	Novel protein_coding	-1.657	0.009
ENSMMUT00000005994	Thromboxane-A synthase	-1.646	0.005
ENSMMUT00000042668	Trefoil factor 1 Precursor	-1.646	0.006
ENSMMUT00000050724	Eukaryotic type signal recognition particle RNA	-1.624	0.004
ENSMMUT00000048192	Novel protein_coding	-1.622	0.009
ENSMMUT00000025083	Doublesex- and mab-3-related transcription factor 3	-1.59	0.009
ENSMMUT00000004286	Protein FAM150A Precursor	-1.585	0.001
ENSMMUT00000025428	Carcinoembryonic antigen-related cell adhesion molecule 4 Precursor	-1.578	0.004
ENSMMUT00000003394	Abhydrolase domain-containing protein 1	-1.569	0.009
ENSMMUT00000007706	Novel protein_coding	-1.559	0.003
ENSMMUT00000009845	Novel protein_coding	-1.553	0.006
ENSMMUT00000020723	Zinc finger protein 409	-1.542	0.009
ENSMMUT00000005890	UPF0417 protein FAM163A	-1.526	0.008
ENSMMUT00000012557	Transmembrane protein 221	-1.504	0.007
ENSMMUT00000030786	Leucine-rich repeat-containing protein 34	-1.503	0.005
ENSMMUT00000039845	Novel protein_coding	-1.487	0.009
ENSMMUT00000015710	Krueppel-like factor 1	-1.435	0.009
ENSMMUT00000025005	Olfactory receptor 5T3	-1.399	0.008
ENSMMUT00000041419	Novel protein_coding	-1.344	0.007
ENSMMUT00000011835	Novel protein_coding	-1.332	0.009
ENSMMUT00000042193	Putative uncharacterized protein IGHEP2 Fragment	-1.282	0.006

[a]positive sign indicates up-regulation while the negative sign represents down-regulation.
