# Supplementary material for: Maternal Bisphenol A Exposure Impacts the Fetal Heart Transcriptome
Source: PLoS One. 2014 Feb 25;9(2):e89096. doi: 10.1371/journal.pone.0089096 (PMC3934879; doi:10.1371/journal.pone.0089096)
Supplement: Table S2 — List of gene transcripts that changed by ≥2 fold (log2 fold change (LFC) = ±1) at p ≤0.01 (unadjusted), in the right ventricle (RV) of the early gestation (EG), maternally BPA exposed vs. matched control, fetuses. (PDF) [file pone.0089096.s002.pdf]

Table S2. List of gene transcripts that changed by ≥ 2 fold ($\log_2$ fold change (LFC) = ± 1) at *p* ≤ 0.01 (unadjusted), in the right ventricle (RV) of the early gestation (EG), maternally BPA exposed *vs.* matched control, fetuses.

SEQ_ID	Gene description	$\log_2$ fold change[a]	*p* value
ENSMMUT00000035850	Y RNA	2.416	0.007
ENSMMUT00000037021	Y RNA	2.383	0
ENSMMUT00000034864	Y RNA	2.375	0.001
ENSMMUT00000050277	Y RNA	2.318	0.006
ENSMMUT00000035315	U6 spliceosomal RNA	2.312	0
ENSMMUT00000034894	Y RNA	2.283	0
ENSMMUT00000036920	mml-mir-554	2.218	0.005
ENSMMUT00000049665	mml-mir-554	2.042	0.006
ENSMMUT00000048496	5S ribosomal RNA	1.991	0.007
ENSMMUT00000021101	Bone morphogenetic protein 10 Precursor	1.981	0.004
ENSMMUT00000036355	Y RNA	1.981	0.005
ENSMMUT00000034209	Y RNA	1.917	0.005
ENSMMUT00000037993	Y RNA	1.914	0.003
ENSMMUT00000036432	U6 spliceosomal RNA	1.888	0.003
ENSMMUT00000035277	Y RNA	1.81	0.001
ENSMMUT00000050574	7SK RNA	1.779	0.004
ENSMMUT00000048813	5S ribosomal RNA	1.715	0.001
ENSMMUT00000049334	NOVEL miRNA	1.7	0.002
ENSMMUT00000037837	Y RNA	1.615	0.005
ENSMMUT00000050646	5S ribosomal RNA	1.608	0
ENSMMUT00000050420	7SK RNA	1.607	0.002
ENSMMUT00000049037	Y RNA	1.601	0.003
ENSMMUT00000036334	7SK RNA	1.595	0.006
ENSMMUT00000037759	U6 spliceosomal RNA	1.579	0.003
ENSMMUT00000038192	Novel SnRNA	1.567	0.002
ENSMMUT00000035250	Y RNA	1.557	0.001
ENSMMUT00000050676	U6 spliceosomal RNA	1.538	0.004
ENSMMUT00000037294	U6 spliceosomal RNA	1.486	0.004
ENSMMUT00000037573	Small nucleolar RNA SNORA70	1.481	0.002
ENSMMUT00000033962	Y RNA	1.476	0.003
ENSMMUT00000036933	mml-mir-644	1.468	0.006
ENSMMUT00000050898	Novel miRNA	1.407	0.004
ENSMMUT00000041981	Novel protein_coding	1.383	0.001
ENSMMUT00000050537	7SK RNA	1.383	0.002
ENSMMUT00000040134	Novel protein_coding	1.362	0.007
ENSMMUT00000037746	U6 spliceosomal RNA	1.343	0.005
ENSMMUT00000034103	U6 spliceosomal RNA	1.314	0.001
ENSMMUT00000033997	Y RNA	1.312	0.009
ENSMMUT00000049493	NOVEL miRNA	1.306	0.009
ENSMMUT00000050700	U6 spliceosomal RNA	1.289	0.006
ENSMMUT00000046159	Novel protein_coding	1.287	0.001
ENSMMUT00000040355	Novel protein_coding	1.263	0.01

ENSMMUT00000038006	Y RNA	1.262	0.008
ENSMMUT00000023817	Novel protein_coding	1.258	0.007
ENSMMUT00000037751	U6 spliceosomal RNA	1.239	0.005
ENSMMUT00000012626	Novel protein_coding	1.224	0.004
ENSMMUT00000046181	Novel protein_coding	1.223	0.009
ENSMMUT00000010802	Novel protein_coding	1.201	0.009
ENSMMUT00000029903	Novel protein_coding	1.193	0.004
ENSMMUT00000039171	Tubulin beta-3 chain	1.179	0.008
ENSMMUT00000008733	Novel protein_coding	1.162	0.007
ENSMMUT00000041728	Novel protein_coding	1.16	0.009
ENSMMUT00000019673	Novel protein_coding	1.155	0.005
ENSMMUT00000041329	Novel protein_coding	1.144	0.006
ENSMMUT00000037656	U6 spliceosomal RNA	1.132	0.004
ENSMMUT00000042878	Novel protein_coding	1.118	0.007
ENSMMUT00000040087	Novel protein_coding	1.113	0.007
ENSMMUT00000014245	Novel protein_coding	1.08	0.007
ENSMMUT00000037625	Novel SnRNA	1.064	0.005
ENSMMUT00000041362	Novel protein_coding	1.053	0.008
ENSMMUT00000018829	Novel protein_coding	1.047	0.008
ENSMMUT00000019583	60S ribosomal protein L9	1.022	0.009
ENSMMUT00000050328	Eukaryotic type signal recognition particle RNA	1.02	0.007
ENSMMUT00000007850	Novel protein_coding	1.019	0.008
ENSMMUT00000033757	Small nucleolar RNA SNORD52	-1.821	0.007
ENSMMUT00000050386	NOVEL miRNA	-1.806	0.002
ENSMMUT00000036703	mml-mir-140	-1.634	0.001
ENSMMUT00000050708	NOVEL miRNA	-1.598	0.005
ENSMMUT00000035869	U6 spliceosomal RNA	-1.598	0.008
ENSMMUT00000036512	Small nucleolar RNA SNORA28	-1.571	0.004
ENSMMUT00000051035	Eukaryotic type signal recognition particle RNA	-1.561	0.002
ENSMMUT00000049348	NOVEL miRNA	-1.477	0.004
ENSMMUT00000049642	NOVEL miRNA	-1.199	0.007
ENSMMUT00000041036	MutS protein homolog 5	-1.176	0.003

[a]positive sign indicates upregulation while the negative sign represents downregulation.
