# Supplementary material for: Maternal Bisphenol A Exposure Impacts the Fetal Heart Transcriptome
Source: PLoS One. 2014 Feb 25;9(2):e89096. doi: 10.1371/journal.pone.0089096 (PMC3934879; doi:10.1371/journal.pone.0089096)
Supplement: Table S3 — List of gene transcripts that changed by ≥2 fold (log2 fold change (LFC) = ±1), at p ≤0.01 (unadjusted), in the left atrium (LA) of the early gestation (EG), maternally BPA exposed vs. matched control, fetuses. (PDF) [file pone.0089096.s003.pdf]

Table S3. List of gene transcripts that changed by ≥ 2 fold (log_2 fold change (LFC) = ± 1), at *p* ≤ 0.01 (unadjusted), in the left atrium (LA) of the early gestation (EG), maternally BPA exposed *vs.* matched control, fetuses.

SEQ_ID	Gene description	log_2 fold change[a]	*p* value
ENSMMUT00000044344	Novel protein_coding	2.784	0.001
ENSMMUT00000003381	Interferon alpha-inducible protein 27-like protein 1	2.597	0
ENSMMUT00000049665	Novel miRNA	2.594	0.004
ENSMMUT00000035933	Y RNA	2.472	0.009
ENSMMUT00000049499	mml-mir-612	2.023	0.001
ENSMMUT00000036774	mml-mir-15a	1.907	0.001
ENSMMUT00000035992	7SK RNA	1.889	0.003
ENSMMUT00000050914	5S ribosomal RNA	1.781	0.009
ENSMMUT00000050648	Eukaryotic type signal recognition particle RNA	1.753	0.006
ENSMMUT00000036703	mml-mir-140	1.72	0.003
ENSMMUT00000050165	Y RNA	1.699	0.005
ENSMMUT00000035621	5S ribosomal RNA	1.648	0.001
ENSMMUT00000032952	Catenin delta-2 (Delta-catenin)	1.579	0.003
ENSMMUT00000034520	Y RNA	1.56	0.002
ENSMMUT00000033591	Y RNA	1.519	0.005
ENSMMUT00000028853	Small conductance calcium-activated potassium channel protein 2	1.427	0.008
ENSMMUT00000035922	U1 spliceosomal RNA	1.412	0.007
ENSMMUT00000050425	Eukaryotic type signal recognition particle RNA	1.297	0.009
ENSMMUT00000018597	Amiloride-sensitive cation channel 4	1.232	0.003
ENSMMUT00000016593	Hexokinase-2	1.185	0.01
ENSMMUT00000050317	7SK RNA	1.165	0.009
ENSMMUT00000051150	U2 spliceosomal RNA	1.082	0.007
ENSMMUT00000005293	Beta-mannosidase Precursor	1.04	0.005
ENSMMUT00000049389	Novel miRNA	-2.812	0
ENSMMUT00000036241	U6 spliceosomal RNA	-2.726	0.001
ENSMMUT00000015266	Fatty acid-binding protein	-2.188	0.002
ENSMMUT00000037756	Small nucleolar RNA SNORD113/SNORD114 family	-1.932	0.001
ENSMMUT00000014502	Myosin-11	-1.928	0.006
ENSMMUT00000036584	mml-mir-346	-1.856	0.003
ENSMMUT00000036714	mml-mir-7-1	-1.693	0.003
ENSMMUT00000035137	7SK RNA	-1.683	0.005
ENSMMUT00000036801	mml-mir-25	-1.664	0.005
ENSMMUT00000050182	U5 spliceosomal RNA	-1.403	0.003
ENSMMUT00000036493	Small nucleolar RNA SNORD18	-1.364	0.006
ENSMMUT00000037449	U6 spliceosomal RNA	-1.338	0.006
ENSMMUT00000035424	U6 spliceosomal RNA	-1.315	0.01
ENSMMUT00000023399	Tumor necrosis factor receptor superfamily member 10A Precursor	-1.309	0.004
ENSMMUT00000040036	Serine/threonine-protein kinase Sgk1	-1.3	0.007
ENSMMUT00000036264	Small nucleolar RNA SNORA62/SNORA6 family	-1.293	0.003
ENSMMUT00000050553	Eukaryotic type signal recognition particle RNA	-1.281	0.008
ENSMMUT00000033983	U6 spliceosomal RNA	-1.255	0.009
ENSMMUT00000037948	U6 spliceosomal RNA	-1.25	0.003
ENSMMUT00000004698	Novel protein_coding	-1.208	0.009

ENSMMUT00000020061	UPF0573 protein C2orf70	-1.201	0.006
ENSMMUT00000005737	Fc receptor-like B Precursor	-1.134	0.002
ENSMMUT00000037508	Y RNA	-1.063	0.009

[a]positive sign indicates upregulation while the negative sign represents downregulation.
