# Supplementary material for: Maternal Bisphenol A Exposure Impacts the Fetal Heart Transcriptome
Source: PLoS One. 2014 Feb 25;9(2):e89096. doi: 10.1371/journal.pone.0089096 (PMC3934879; doi:10.1371/journal.pone.0089096)
Supplement: Table S4 — List of gene transcripts that changed by ≥2 fold (log2 fold change (LFC) = ±1), at p ≤0.01 (unadjusted), in the right atrium (RA) of the early gestation (EG), maternally BPA exposed vs. matched control, fetuses. (PDF) [file pone.0089096.s004.pdf]

Table S4. List of gene transcripts that changed by ≥ 2 fold (log_2 fold change (LFC) = ± 1), at *p* ≤ 0.01 (unadjusted), in the right atrium (RA) of the early gestation (EG), maternally BPA exposed *vs.* matched control, fetuses.

SEQ_ID	Gene description	log_2 fold change[a]	*p* value
ENSMMUT00000020971	Seven transmembrane helix receptor	3.096	0.002
ENSMMUT00000003381	Interferon alpha-inducible protein 27-like protein 1	2.359	0.001
ENSMMUT00000005370	Beta-defensin 1 precursor	1.651	0.003
ENSMMUT00000024823	Myosin-XVI	1.555	0.001
ENSMMUT00000034493	Y RNA	1.529	0.006
ENSMMUT00000042221	Dehydrogenase/reductase SDR family member 7C Precursor	1.517	0.004
ENSMMUT00000033771	U6 spliceosomal RNA	-2.792	0.005
ENSMMUT00000036920	mml-mir-554	-2.565	0.005
ENSMMUT00000033615	U6 spliceosomal RNA	-1.966	0.001
ENSMMUT00000037500	U6 spliceosomal RNA	-1.87	0.001
ENSMMUT00000034045	U6 spliceosomal RNA	-1.7	0.005
ENSMMUT00000034747	Small nucleolar RNA SNORA58	-1.593	0.008
ENSMMUT00000030838	Leucine-rich repeat-containing protein 7	-1.548	0.006

[a]positive sign indicates upregulation while the negative sign represents downregulation.
