# Supplementary material for: Maternal Bisphenol A Exposure Impacts the Fetal Heart Transcriptome
Source: PLoS One. 2014 Feb 25;9(2):e89096. doi: 10.1371/journal.pone.0089096 (PMC3934879; doi:10.1371/journal.pone.0089096)
Supplement: Table S5 — List of gene transcripts that changed by ≥2 fold (log2 fold change (LFC) = ±1), at p ≤0.01 (unadjusted), in the left ventricle (LV) of the late gestation (LG), maternally BPA exposed vs. matched control, fetuses. (PDF) [file pone.0089096.s005.pdf]

Table S5. List of gene transcripts that changed by ≥ 2 fold (log_2 fold change (LFC) = ± 1), at *p* ≤ 0.01 (unadjusted), in the left ventricle (LV) of the late gestation (LG), maternally BPA exposed *vs.* matched control, fetuses.

SEQ_ID	Gene description	log_2 fold change[a]	*p* value
ENSMMUT00000034332	U1 spliceosomal RNA	4.636	0
ENSMMUT00000050726	U6 spliceosomal RNA	4.351	0.003
ENSMMUT00000037441	U6 spliceosomal RNA	4.211	0.006
ENSMMUT00000035933	Y RNA	4.143	0.001
ENSMMUT00000036578	mml-mir-224	4.093	0
ENSMMUT00000048847	Small nucleolar RNA SNORD115	4.09	0.005
ENSMMUT00000038016	5S ribosomal RNA	4.065	0
ENSMMUT00000035066	U6 spliceosomal RNA	3.978	0.004
ENSMMUT00000033742	Small nucleolar RNA SNORA51	3.934	0.004
ENSMMUT00000048818	Small nucleolar RNA SNORD96 family	3.867	0.002
ENSMMUT00000037540	U6 spliceosomal RNA	3.818	0.008
ENSMMUT00000034541	U6 spliceosomal RNA	3.732	0.009
ENSMMUT00000048554	Novel miRNA	3.631	0.008
ENSMMUT00000035721	Small nucleolar RNA SNORD50	3.366	0.007
ENSMMUT00000036316	Y RNA	3.268	0.005
ENSMMUT00000051106	U6 spliceosomal RNA	3.205	0.006
ENSMMUT00000034544	5S ribosomal RNA	3.199	0.003
ENSMMUT00000043960	Postacrosomal sheath WW domain-binding protein	3.082	0.001
ENSMMUT00000035318	Small nucleolar RNA SNORA71	3.08	0.005
ENSMMUT00000049550	Small nucleolar RNA SNORA3/SNORA45 family	3.063	0.005
ENSMMUT00000037632	U4 spliceosomal RNA	2.938	0.005
ENSMMUT00000050617	Eukaryotic type signal recognition particle RNA	2.905	0.003
ENSMMUT00000037000	mml-mir-562	2.896	0.009
ENSMMUT00000035905	Small nucleolar RNA SNORD42	2.871	0.003
ENSMMUT00000048896	5S ribosomal RNA	2.817	0.006
ENSMMUT00000033750	5S ribosomal RNA	2.779	0
ENSMMUT00000050214	Eukaryotic type signal recognition particle RNA	2.692	0.005
ENSMMUT00000034178	U6 spliceosomal RNA	2.687	0.003
ENSMMUT00000036624	mml-mir-205	2.583	0.001
ENSMMUT00000036351	5S ribosomal RNA	2.575	0.006
ENSMMUT00000040661	Novel protein_coding	2.554	0.005
ENSMMUT00000001811	Olfactory receptor 1M1	2.478	0.002
ENSMMUT00000036089	Y RNA	2.47	0.004
ENSMMUT00000050254	Eukaryotic type signal recognition particle RNA	2.437	0.006
ENSMMUT00000050805	Eukaryotic type signal recognition particle RNA	2.4	0.001
ENSMMUT00000023185	Novel protein_coding	2.399	0.007
ENSMMUT00000050913	Eukaryotic type signal recognition particle RNA	2.391	0.001
ENSMMUT00000044775	Protein GDF5OS, mitochondrial Precursor	2.361	0.002
ENSMMUT00000024211	Pancreatic secretory trypsin inhibitor Precursor	2.359	0.004
ENSMMUT00000050347	Eukaryotic type signal recognition particle RNA	2.296	0.002
ENSMMUT00000050965	Novel miRNA	2.278	0.001
ENSMMUT00000030639	Beta-1,3-galactosyltransferase 1	2.277	0.001

ENSMMUT00000048576	U6 spliceosomal RNA	2.198	0.01
ENSMMUT00000050226	7SK RNA	2.162	0.001
ENSMMUT00000031246	Src kinase-associated phosphoprotein 1	2.149	0.005
ENSMMUT00000039524	T-cell leukemia/lymphoma protein 1A	2.053	0.005
ENSMMUT00000041515	Novel protein_coding	2.039	0.001
ENSMMUT00000011918	Interferon-induced protein with tetratricopeptide repeats 1-like protein	2.03	0.007
ENSMMUT00000038748	Novel SnRNA	2.015	0
ENSMMUT00000035667	U4 spliceosomal RNA	2.007	0.009
ENSMMUT00000014742	Novel protein_coding	1.957	0.007
ENSMMUT00000012793	Alpha-tocopherol transfer protein	1.946	0.006
ENSMMUT00000003461	Phosphoethanolamine/phosphocholine phosphatase	1.937	0.002
ENSMMUT00000025736	Retinoic acid receptor responder protein 3	1.909	0.003
ENSMMUT00000003633	Novel protein_coding	1.871	0.007
ENSMMUT00000014568	Uncharacterized protein C1orf168	1.856	0.001
ENSMMUT00000004020	Ganglioside-induced differentiation-associated protein 1-like 1	1.834	0.005
ENSMMUT00000013014	Myelin transcription factor 1-like protein	1.795	0.003
ENSMMUT00000035351	Small nucleolar RNA SNORA5	1.79	0.005
ENSMMUT00000025577	Novel protein_coding	1.714	0.008
ENSMMUT00000013487	ADAM 12 Precursor	1.678	0.001
ENSMMUT00000017238	Novel protein_coding	1.666	0.002
ENSMMUT00000037794	U6 spliceosomal RNA	1.631	0.005
ENSMMUT00000018498	3-oxo-5-alpha-steroid 4-dehydrogenase 1	1.593	0.006
ENSMMUT00000015495	ADAM 21 Precursor	1.561	0.01
ENSMMUT00000000268	chemokine CCL28/MEC	1.557	0.01
ENSMMUT00000018059	Copper-transporting ATPase 2	1.544	0.005
ENSMMUT00000027913	Citron Rho-interacting kinase	1.524	0.006
ENSMMUT00000018826	Novel protein_coding	1.508	0.006
ENSMMUT00000031618	Coiled-coil domain-containing protein 129	1.471	0.008
ENSMMUT00000026179	NIPA-like protein 1	1.45	0.003
ENSMMUT00000000295	Prominin-2 Precursor	1.433	0.005
ENSMMUT00000014527	Adenylate cyclase type 10	1.418	0.004
ENSMMUT00000022103	Novel protein_coding	1.378	0.007
ENSMMUT00000042193	Putative uncharacterized protein IGHEP2 Fragment	1.317	0.005
ENSMMUT00000014015	Carcinoembryonic antigen-related cell adhesion molecule 16 Precursor	1.245	0.009
ENSMMUT00000010814	Hemopexin Precursor	1.232	0.008
ENSMMUT00000048699	Small nucleolar RNA SNORD81	-5.685	0.006
ENSMMUT00000020702	Myosin-6	-5.385	0.004
ENSMMUT00000048424	Novel miRNA	-4.723	0
ENSMMUT00000035190	Y RNA	-4.248	0.005
ENSMMUT00000036907	mml-mir-133c	-4.084	0.004
ENSMMUT00000050085	5S ribosomal RNA	-4.068	0.007
ENSMMUT00000037542	Y RNA	-4.062	0
ENSMMUT00000048980	mml-mir-487a	-4.007	0.001
ENSMMUT00000051032	7SK RNA	-3.401	0.008
ENSMMUT00000048952	U6 spliceosomal RNA	-3.4	0.002
ENSMMUT00000034845	Y RNA	-3.245	0.002
ENSMMUT00000005248	Family 13, member C1-like protein	-3.159	0.001

ENSMMUT00000050994	Eukaryotic type signal recognition particle RNA	-3.123	0
ENSMMUT00000041184	Novel protein_coding	-3.123	0.004
ENSMMUT00000050174	Eukaryotic type signal recognition particle RNA	-2.918	0.003
ENSMMUT00000036552	mml-mir-184	-2.875	0.01
ENSMMUT00000012954	Kinesin heavy chain isoform 5C	-2.837	0.009
ENSMMUT00000031937	Novel protein_coding	-2.836	0.003
ENSMMUT00000036964	mml-mir-640	-2.814	0.006
ENSMMUT00000015490	Receptor-type tyrosine-protein phosphatase R Precursor	-2.716	0.002
ENSMMUT00000014606	Adenylate cyclase type 8	-2.713	0.001
ENSMMUT00000024519	Secretogranin-3 Precursor	-2.664	0.003
ENSMMUT00000003632	Doublesex- and mab-3-related transcription factor A1	-2.627	0.001
ENSMMUT00000009882	Neutral ceramidase	-2.619	0.002
ENSMMUT00000009386	Sodium/bile acid cotransporter 4	-2.558	0.009
ENSMMUT00000022875	Try4.	-2.521	0.002
ENSMMUT00000014748	Novel protein_coding	-2.509	0.001
ENSMMUT00000050648	Eukaryotic type signal recognition particle RNA	-2.504	0.002
ENSMMUT00000032457	Homeobox protein goosecoid	-2.489	0.003
ENSMMUT00000035521	Y RNA	-2.479	0.004
ENSMMUT00000001371	Novel protein_coding	-2.43	0.006
ENSMMUT00000037901	Y RNA	-2.386	0.004
ENSMMUT00000000042	Transmembrane protein 132C Precursor	-2.36	0.009
ENSMMUT00000009956	Novel protein_coding	-2.335	0.001
ENSMMUT00000006600	Putative uncharacterized serine/threonine-protein kinase SgK069	-2.335	0.008
ENSMMUT00000002996	Transcription factor LBX2	-2.328	0
ENSMMUT00000006112	EF-hand domain-containing protein LOC100130771	-2.294	0.002
ENSMMUT00000009836	HIP14-related protein (Fragment).	-2.276	0.007
ENSMMUT00000050736	U4 spliceosomal RNA	-2.255	0.007
ENSMMUT00000001109	Novel SnRNA	-2.19	0.001
ENSMMUT00000049861	Novel miRNA	-2.163	0.007
ENSMMUT00000008363	Novel protein_coding	-2.133	0.005
ENSMMUT00000049990	7SK RNA	-2.125	0.007
ENSMMUT00000021952	2-acylglycerol O-acyltransferase 1	-2.118	0.004
ENSMMUT00000006058	Inactive phospholipase D5	-2.074	0.002
ENSMMUT00000033112	Novel protein_coding	-1.999	0.004
ENSMMUT00000022683	Mas-related G-protein coupled receptor member X2.	-1.999	0.008
ENSMMUT00000028428	Dual specificity protein kinase TTK	-1.988	0.005
ENSMMUT00000026374	Novel protein_coding	-1.887	0.008
ENSMMUT00000007484	ADAM 28 Precursor	-1.886	0.001
ENSMMUT00000014836	WNT1-inducible-signaling pathway protein 2 Precursor	-1.873	0.002
ENSMMUT00000043082	Novel protein_coding	-1.814	0.004
ENSMMUT00000045799	Endothelin-converting enzyme 2	-1.811	0.003
ENSMMUT00000037517	U6 spliceosomal RNA	-1.79	0.005
ENSMMUT00000012608	M-phase inducer phosphatase 3	-1.738	0.003
ENSMMUT00000003564	Spermatogenesis-associated protein 2-like protein	-1.738	0.01
ENSMMUT00000019642	Regulator of G-protein signaling 18	-1.716	0.006
ENSMMUT00000026823	Centromere-associated protein E	-1.648	0.002
ENSMMUT00000026779	Novel protein_coding	-1.581	0.002

ENSMMUT00000021761	Serine/threonine-protein kinase LMTK1	-1.567	0.007
ENSMMUT00000027726	Dihydropyrimidinase-related protein 5	-1.55	0.003
ENSMMUT00000032215	Potassium voltage-gated channel subfamily A member 5	-1.489	0.01
ENSMMUT00000045794	Ankyrin repeat and sterile alpha motif domain-containing protein 1B	-1.483	0.008
ENSMMUT00000024250	Spermatogenesis-associated protein 20 Precursor	-1.425	0.005
ENSMMUT00000023031	Chondromodulin-1 Precursor	-1.389	0.005
ENSMMUT00000001156	KDEL motif-containing protein 2 Precursor	-1.338	0.01
ENSMMUT00000014365	Neutral amino acid transporter A	-1.295	0.01
ENSMMUT00000045163	Myosin-IIIB	-1.29	0.007
ENSMMUT00000032258	Aquaporin-2	-1.285	0.009
ENSMMUT00000002771	Solute carrier family 25 member 33	-1.257	0.008
ENSMMUT00000008173	Novel protein_coding	-1.149	0.009

[a]positive sign indicates upregulation while the negative sign represents downregulation.
