# Supplementary material for: Maternal Bisphenol A Exposure Impacts the Fetal Heart Transcriptome
Source: PLoS One. 2014 Feb 25;9(2):e89096. doi: 10.1371/journal.pone.0089096 (PMC3934879; doi:10.1371/journal.pone.0089096)
Supplement: Table S6 — List of gene transcripts that changed by ≥2 fold (log2 fold change (LFC) = ±1), at p ≤0.01 (unadjusted), in the right ventricle (RV) of the late gestation (LG), maternally BPA exposed vs. matched control, fetuses. (PDF) [file pone.0089096.s006.pdf]

Table S6. List of gene transcripts that changed by ≥ 2 fold (log_2 fold change (LFC) = ± 1), at *p* ≤ 0.01 (unadjusted), in the right ventricle (RV) of the late gestation (LG), maternally BPA exposed *vs.* matched control, fetuses.

SEQ_ID	Gene description	log_2 fold change[a]	*p* value
ENSMMUT00000049708	Novel miRNA	2.342	0.006
ENSMMUT00000013118	Cellular retinoic acid-binding protein 1	2.24	0.004
ENSMMUT00000037347	U6 spliceosomal RNA	2.173	0.003
ENSMMUT00000049023	Small nucleolar RNA SNORD77	2.038	0
ENSMMUT00000036839	Novel miRNA	2.025	0.002
ENSMMUT00000048784	Small nucleolar RNA SNORD69 [Source: RFAM 9.0]	1.938	0
ENSMMUT00000050311	7SK RNA	1.779	0.003
ENSMMUT00000048702	U6 spliceosomal RNA	1.74	0.002
ENSMMUT00000035273	Y RNA	1.704	0.008
ENSMMUT00000048412	Novel miRNA	1.594	0.001
ENSMMUT00000036645	mml-mir-197	1.573	0.008
ENSMMUT00000023702	EGF-containing fibulin-like extracellular matrix protein 1 Precursor	1.551	0.005
ENSMMUT00000035550	Y RNA	1.526	0.005
ENSMMUT00000050957	Small nucleolar RNA SNORD113/SNORD114 family	1.483	0.002
ENSMMUT00000034008	U4 spliceosomal RNA	1.43	0.002
ENSMMUT00000014881	Netrin receptor UNC5D Precursor	1.415	0.009
ENSMMUT00000050843	Novel miRNA	1.404	0.009
ENSMMUT00000033627	Small nucleolar RNA SNORD113/SNORD114 family	1.387	0.003
ENSMMUT00000049722	Novel miRNA	1.377	0.002
ENSMMUT00000037303	U6 spliceosomal RNA	1.351	0.002
ENSMMUT00000013597	Transcription regulator protein BACH2	1.332	0.006
ENSMMUT00000035059	Small nucleolar RNA SNORA25	1.318	0.01
ENSMMUT00000002406	Novel protein_coding	1.239	0.005
ENSMMUT00000013487	ADAM 12 Precursor	1.213	0.005
ENSMMUT00000043921	Novel protein_coding	1.21	0.002
ENSMMUT00000008957	Sugar phosphate exchanger 2	1.202	0.009
ENSMMUT00000024326	Transmembrane 4 L6 family member 19	1.15	0.009
ENSMMUT00000048433	U6 spliceosomal RNA	1.149	0.006
ENSMMUT00000045352	Probable G-protein coupled receptor 133 Precursor	1.125	0.008
ENSMMUT00000050204	Eukaryotic type signal recognition particle RNA	1.05	0.006
ENSMMUT00000031900	Probable serine protease HTRA3 Precursor	1.026	0.01
ENSMMUT00000036785	mml-mir-452	-2.664	0.003
ENSMMUT00000050571	U6 spliceosomal RNA	-2.514	0
ENSMMUT00000046184	Novel protein_coding	-2.302	0.009
ENSMMUT00000036588	mml-mir-18	-2.282	0.01
ENSMMUT00000037517	U6 spliceosomal RNA	-2.247	0.002
ENSMMUT00000034361	U6 spliceosomal RNA	-2.114	0.002
ENSMMUT00000048719	mml-mir-518c	-2.092	0.003
ENSMMUT00000035807	U6 spliceosomal RNA	-1.986	0.002
ENSMMUT00000037438	U6 spliceosomal RNA	-1.88	0.007
ENSMMUT00000033835	U6 spliceosomal RNA	-1.828	0.003
ENSMMUT00000037484	Small nucleolar RNA SNORD115	-1.821	0.004
ENSMMUT00000037709	U6 spliceosomal RNA	-1.789	0.004

ENSMMUT00000035165	U6 spliceosomal RNA	-1.684	0.003
ENSMMUT00000050117	U6 spliceosomal RNA	-1.655	0.005
ENSMMUT00000038068	Y RNA	-1.589	0.001
ENSMMUT00000036621	mml-mir-16-2	-1.58	0.002
ENSMMUT00000036374	Small nucleolar RNA SNORD45	-1.538	0.006
ENSMMUT00000051013	5S ribosomal RNA	-1.511	0.008
ENSMMUT00000048678	U6 spliceosomal RNA	-1.479	0.009
ENSMMUT00000033672	U6 spliceosomal RNA	-1.445	0.009
ENSMMUT00000034441	U6 spliceosomal RNA	-1.406	0.008
ENSMMUT00000033856	U6 spliceosomal RNA	-1.405	0.002
ENSMMUT00000022906	Novel protein_coding	-1.388	0.005
ENSMMUT00000048513	U6 spliceosomal RNA	-1.358	0.009
ENSMMUT00000003324	Novel protein_coding	-1.346	0.003
ENSMMUT00000050842	7SK RNA	-1.342	0.006
ENSMMUT00000008996	Transmembrane protein FAM155B	-1.303	0.007
ENSMMUT00000035047	Small nucleolar RNA SNORD74	-1.256	0.008
ENSMMUT00000051082	7SK RNA	-1.234	0.003
ENSMMUT00000008950	Novel protein_coding	-1.189	0.003
ENSMMUT00000034391	Y RNA	-1.176	0.005
ENSMMUT00000015667	Novel protein_coding	-1.124	0.005
ENSMMUT00000050288	5S ribosomal RNA	-1.118	0.008
ENSMMUT00000037617	Y RNA	-1.112	0.01
ENSMMUT00000032206	Eukaryotic translation initiation factor 5A-2	-1.074	0.004

[a]positive sign indicates upregulation while the negative sign represents downregulation.
