# Supplementary material for: Maternal Bisphenol A Exposure Impacts the Fetal Heart Transcriptome
Source: PLoS One. 2014 Feb 25;9(2):e89096. doi: 10.1371/journal.pone.0089096 (PMC3934879; doi:10.1371/journal.pone.0089096)
Supplement: Table S7 — List of gene transcripts that changed by ≥2 fold (log2 fold change (LFC) = ±1), at p ≤0.01 (unadjusted), in the left atrium (LA) of the late gestation (LG), maternally BPA exposed vs. matched control, fetuses. (PDF) [file pone.0089096.s007.pdf]

Table S7. List of gene transcripts that changed by ≥ 2 fold (log_2 fold change (LFC) = ± 1), at p ≤ 0.01 (unadjusted), in the left atrium (LA) of the late gestation (LG), maternally BPA exposed *vs.* matched control, fetuses.

SEQ_ID	Gene description	log_2 fold change[a]	p value
ENSMMUT00000011786	5-aminolevulinate synthase, erythroid-specific, mitochondrial Precursor	2.807	0.005
ENSMMUT00000050668	Y RNA	2.226	0.002
ENSMMUT00000035137	7SK RNA	2.141	0.001
ENSMMUT00000020210	Novel protein_coding	2.1	0.001
ENSMMUT00000014314	CAMPATH-1 antigen Precursor	2.078	0.009
ENSMMUT00000048610	mml-mir-507	2.028	0.006
ENSMMUT00000050624	5S ribosomal RNA	1.902	0
ENSMMUT00000011955	Contactin-6 Precursor	1.88	0.001
ENSMMUT00000038412	Novel protein_coding	1.863	0.003
ENSMMUT00000034030	U6 spliceosomal RNA	1.846	0.006
ENSMMUT00000028746	Granulocyte colony-stimulating factor receptor Precursor	1.782	0.009
ENSMMUT00000005267	Novel protein_coding	1.756	0.007
ENSMMUT00000043987	MHC class I antigen	1.743	0.002
ENSMMUT00000031238	N-acetyllactosaminide beta-1,6-N-acetylglucosaminyl-transferase	1.732	0.002
ENSMMUT00000031333	Acyloxyacyl hydrolase Precursor	1.728	0.006
ENSMMUT00000037064	Small nucleolar RNA Z17	1.715	0.001
ENSMMUT00000050758	Small nucleolar RNA SNORD41	1.695	0.01
ENSMMUT00000034881	Small nucleolar RNA SNORA9	1.667	0.006
ENSMMUT00000050939	Eukaryotic type signal recognition particle RNA	1.658	0.003
ENSMMUT00000005345	Low affinity immunoglobulin gamma Fc region receptor II-b Precursor	1.652	0.002
ENSMMUT00000015749	Novel protein_coding	1.639	0
ENSMMUT00000015593	Novel protein_coding	1.572	0.009
ENSMMUT00000035936	Y RNA	1.522	0.008
ENSMMUT00000048869	U6 spliceosomal RNA	1.514	0.006
ENSMMUT00000007658	ELMO domain-containing protein 1	1.507	0.006
ENSMMUT00000015741	Alcohol dehydrogenase class 4 mu/sigma chain	1.5	0.001
ENSMMUT00000033373	Novel protein_coding	1.488	0.004
ENSMMUT00000049835	Novel miRNA	1.425	0.005
ENSMMUT00000048679	Small nucleolar RNA SNORA48	1.378	0.009
ENSMMUT00000048568	Small nucleolar RNA SNORD72	1.376	0.004
ENSMMUT00000007835	Collagen alpha-1(XIX) chain Precursor	1.373	0.005
ENSMMUT00000032233	Platelet-derived growth factor C Precursor	1.349	0.007
ENSMMUT00000013272	Diphosphoinositol polyphosphate phosphohydrolase 3-beta	1.309	0.001
ENSMMUT00000050330	Eukaryotic type signal recognition particle RNA	1.309	0.004
ENSMMUT00000010940	Paired mesoderm homeobox protein 2B	1.308	0.002
ENSMMUT00000013515	Complement factor D Precursor	1.299	0.005
ENSMMUT00000014921	Cathepsin S Precursor	1.254	0.004
ENSMMUT00000043889	Tripartite motif-containing protein 34	1.236	0.002
ENSMMUT00000017316	Cytokine receptor common subunit beta Precursor	1.236	0.009
ENSMMUT00000036512	Small nucleolar RNA SNORA28	1.217	0.007
ENSMMUT00000048923	5S ribosomal RNA	1.214	0.003
ENSMMUT00000028364	Novel protein_coding	1.214	0.007

ENSMMUT00000019861	Connective tissue growth factor Precursor	1.21	0.006
ENSMMUT00000019827	Lymphocyte cytosolic protein 2	1.173	0.008
ENSMMUT00000032305	Amyloid beta A4 precursor protein-binding family B member 1-interacting protein	1.156	0.008
ENSMMUT00000029619	Putative uncharacterized protein CD177P Fragment	1.129	0.007
ENSMMUT00000044475	Novel protein_coding	1.116	0.008
ENSMMUT00000050671	Eukaryotic type signal recognition particle RNA	1.084	0.008
ENSMMUT00000050338	U6 spliceosomal RNA	-2.347	0.002
ENSMMUT00000038127	Y RNA	-2.225	0.005
ENSMMUT00000050906	Novel miRNA	-2.208	0.003
ENSMMUT00000050386	Novel miRNA	-1.936	0.002
ENSMMUT00000048826	U6 spliceosomal RNA	-1.931	0.003
ENSMMUT00000036386	5S ribosomal RNA	-1.906	0.002
ENSMMUT00000049261	Small nucleolar RNA SNORA77	-1.865	0.003
ENSMMUT00000049359	Novel miRNA	-1.813	0.007
ENSMMUT00000050945	Novel miRNA	-1.734	0.002
ENSMMUT00000037907	U6 spliceosomal RNA	-1.67	0.002
ENSMMUT00000034615	U6 spliceosomal RNA	-1.582	0.01
ENSMMUT00000035165	U6 spliceosomal RNA	-1.581	0.003
ENSMMUT00000033983	U6 spliceosomal RNA	-1.535	0.002
ENSMMUT00000048404	mml-mir-765	-1.517	0.003
ENSMMUT00000048423	Small nucleolar RNA SNORD78	-1.475	0.001
ENSMMUT00000038278	NovelMt_tRNA	-1.461	0.002
ENSMMUT00000050317	7SK RNA	-1.454	0.002
ENSMMUT00000049446	Novel miRNA	-1.439	0.003
ENSMMUT00000034503	Y RNA	-1.377	0.006
ENSMMUT00000035779	Small nucleolar RNA SNORA25	-1.372	0.006
ENSMMUT00000006769	Multiple C2 and transmembrane domain-containing protein 2	-1.333	0.009
ENSMMUT00000038105	U6 spliceosomal RNA	-1.312	0.003
ENSMMUT00000003143	Rap1 GTPase-activating protein 1	-1.293	0.001
ENSMMUT00000005042	Proline-serine-threonine phosphatase-interacting protein 2	-1.283	0.01
ENSMMUT00000035804	U1 spliceosomal RNA	-1.283	0.01
ENSMMUT00000051074	Eukaryotic type signal recognition particle RNA	-1.226	0.005
ENSMMUT00000016326	Probable G-protein coupled receptor 20	-1.206	0.006
ENSMMUT00000050194	U6 spliceosomal RNA	-1.205	0.008
ENSMMUT00000035968	U6 spliceosomal RNA	-1.202	0.009
ENSMMUT00000048513	U6 spliceosomal RNA	-1.193	0.002
ENSMMUT00000024480	IQ and AAA domain-containing protein	-1.181	0.001
ENSMMUT00000037648	U6 spliceosomal RNA	-1.167	0.007
ENSMMUT00000029589	Novel protein_coding	-1.153	0.003
ENSMMUT00000010292	Copine-6	-1.123	0.007
ENSMMUT00000050498	U4 spliceosomal RNA	-1.11	0.009
ENSMMUT00000037760	5S ribosomal RNA	-1.106	0.004
ENSMMUT00000049987	Eukaryotic type signal recognition particle RNA	-1.099	0.007

[a]positive sign indicates upregulation while the negative sign represents downregulation.
