# Supplementary material for: Maternal Bisphenol A Exposure Impacts the Fetal Heart Transcriptome
Source: PLoS One. 2014 Feb 25;9(2):e89096. doi: 10.1371/journal.pone.0089096 (PMC3934879; doi:10.1371/journal.pone.0089096)
Supplement: Table S8 — List of gene transcripts that changed by ≥2 fold (log2 fold change (LFC) = ±1), at p ≤0.01 (unadjusted), in the right atrium (RA) of the late gestation (LG), maternally BPA exposed vs. matched control, fetuses. (PDF) [file pone.0089096.s008.pdf]

Table S8. List of gene transcripts that changed by ≥ 2 fold (log_2 fold change (LFC) = ± 1), at *p* ≤ 0.01 (unadjusted), in the right atrium (RA) of the late gestation (LG), maternally BPA exposed *vs.* matched control, fetuses.

SEQ_ID	Gene description	log_2 fold change[a]	*p* value
ENSMMUT00000038087	U6 spliceosomal RNA	2.246	0.001
ENSMMUT00000046606	Nuclear receptor subfamily 4 group A member 1	1.816	0.006
ENSMMUT00000035618	U6 spliceosomal RNA	1.738	0.005
ENSMMUT00000035881	Small nucleolar RNA R38	1.598	0.002
ENSMMUT00000009347	Apolipoprotein L domain-containing protein 1	1.535	0.004
ENSMMUT00000036761	mml-mir-20a	-2.109	0.009
ENSMMUT00000035944	U6 spliceosomal RNA	-2.033	0.003
ENSMMUT00000034556	U6 spliceosomal RNA	-1.84	0
ENSMMUT00000035056	U6 spliceosomal RNA	-1.744	0.007
ENSMMUT00000037079	U6 spliceosomal RNA	-1.663	0
ENSMMUT00000037889	U6 spliceosomal RNA	-1.656	0.01

[a]positive sign indicates upregulation while the negative sign represents downregulation.
